# Metabolomics of the Tumor Microenvironment in Pediatric Acute Lymphoblastic Leukemia

**DOI:** 10.1371/journal.pone.0082859

**Published:** 2013-12-13

**Authors:** Stefano Tiziani, Yunyi Kang, Ricky Harjanto, Joshua Axelrod, Carlo Piermarocchi, William Roberts, Giovanni Paternostro

**Affiliations:** 1 Sanford-Burnham Medical Research Institute, La Jolla, California, United States of America; 2 Department of Physics and Astronomy, Michigan State University, East Lansing, Michigan, United States of America; 3 Rady Children’s Hospital, Department of Pediatrics, University of California San Diego, San Diego, California, United States of America; 4 Department of Nutritional Sciences, Dell Pediatric Research Institute, University of Texas at Austin, Austin, Texas, United States of America; Westmead Millennium Institute, University of Sydney, Australia

## Abstract

The tumor microenvironment is emerging as an important therapeutic target. Most studies, however, are focused on the protein components, and relatively little is known of how the microenvironmental metabolome might influence tumor survival. In this study, we examined the metabolic profiles of paired bone marrow (BM) and peripheral blood (PB) samples from 10 children with acute lymphoblastic leukemia (ALL). BM and PB samples from the same patient were collected at the time of diagnosis and after 29 days of induction therapy, at which point all patients were in remission. We employed two analytical platforms, high-resolution magnetic resonance spectroscopy and gas chromatography-mass spectrometry, to identify and quantify 102 metabolites in the BM and PB. Standard ALL therapy, which includes l-asparaginase, completely removed circulating asparagine, but not glutamine. Statistical analyses of metabolite correlations and network reconstructions showed that the untreated BM microenvironment was characterized by a significant network-level signature: a cluster of highly correlated lipids and metabolites involved in lipid metabolism (p<0.006). In contrast, the strongest correlations in the BM upon remission were observed among amino acid metabolites and derivatives (p<9.2×10^-10^). This study provides evidence that metabolic characterization of the cancer niche could generate new hypotheses for the development of cancer therapies.

## Introduction

 Cancer is the leading cause of disease-related death in children, and the most common pediatric cancer is acute lymphoblastic leukemia (ALL)[1]. ALL is an aggressive disease characterized by the accumulation of immature lymphoid cells in the bone marrow (BM) and peripheral blood (PB). Despite marked improvement in treatment, a substantial number of children with ALL die of the disease[2–5]. Moreover, even children who achieve a cure must undergo a long treatment course accompanied by major discomfort and potentially severe side effects[6].

It is now well-established that cancer development, progression, and response to therapy are strongly influenced by the stromal cells, matrix proteins, and secreted molecules that make up the tumor microenvironment[7–9]. Many studies have focused on the protein components of the microenvironment, but relatively little is known of how the local metabolome might influence the course of disease and the tumor response to therapy. Because a unique shift in metabolic phenotype is one of the hallmarks of cancer[10–12], metabolic profiling represents a powerful, and now technically feasible, method to monitor dynamic changes in tumor metabolism over the course of the disease and in response to therapy. Moreover, fluctuations in local metabolite concentrations, especially glucose, fatty acids, and amino acids, have been shown to influence the efficacy of chemotherapy in human cancers[13,14]. Interestingly, ALL cells display a particular dependence on exogenous asparagine for replication, a fact that has been exploited in designing drug treatment regimens. Thus, l-asparaginase, which deaminates circulating asparagine, and, to a lesser extent, glutamine, is a component of the standard chemotherapeutic regimen to treat pediatric ALL[15–19]. Metabolomics could therefore be used to determine whether individual cancers are dependent on particular metabolic pathways, which could then be exploited in designing more targeted cancer therapies [20]. 

Another area in which metabolic profiling of tumors has become increasingly important is in the identification of biomarkers for personalized treatment strategies. Several recent studies have highlighted the diagnostic and the prognostic potential of metabolite profiling in a range of human diseases[20–24], including hematological malignancies such as multiple myeloma[25] and chronic lymphocytic leukemia[26]. Although metabolite analysis is often performed on PB, circulating metabolite concentrations reflect whole body responses to disease and/or therapy. Thus, it is important to recognize that analysis of biofluids at the specific tumor niche is likely to yield more accurate and clinically useful information about the metabolic demands of tumors and could identify novel pharmacodynamic biomarkers to assess the tumor response to therapy.

 In this study, we sought to examine the BM and PB metabolomes of 10 children with pediatric ALL. Paired PB and BM samples were collected from patients at the time of diagnosis and again after 4 weeks of induction therapy, at which point all patients were in disease remission. We analyzed the absolute levels of metabolites and differences between the BM and PB compartments within the same patient, which allowed us to accurately assess the effects of tumor burden and induction therapy on the respective metabolomes. Because the BM of ALL patients is almost completely invaded with cancer cells at the time of diagnosis, and numerous organs contribute to the metabolic content of PB, analysis of BM samples may provide critical information not captured by analysis of plasma samples. In this regard, the leukemic BM and PB microenvironments show many metabolic differences[27,28], including lower oxygen tension in the BM. 

We employed two analytical platforms, high-resolution magnetic resonance spectroscopy (MRS) and gas chromatography-mass spectrometry (GC-MS), to generate a large metabolomics dataset profiling the cancer niche and PB before therapy and after remission. We used multivariate statistical analysis techniques to compare metabolomic profiles and univariate analysis to compare changes in individual metabolites. We identified and quantified 102 metabolites that reveal a clear switch in the balance between lipid and amino acid metabolism in tumor-burdened versus tumor-free BM. 

Obtaining metabolomic samples of the cancer niche and peripheral blood from the same patient before therapy and after remission is a novel approach for ALL and for cancer metabolism in general. The datasets generated in this study represent a unique clinical resource that complements other experimental approaches to cancer metabolism. 

## Methods

### Declaration of ethical approval

All clinical investigations were conducted according to Declaration of Helsinki principles. All human studies were approved by the UCSD Human Research Protections Programs IRB. Written informed consent was received from participants prior to inclusion in the study. Written informed consent and parental permission were obtained in accordance with Institutional Review Board guidelines.

### Patient characteristics and sample collection

Paired BM and PB specimens from 10 children diagnosed with B-ALL were collected at the Rady Children’s Hospital (San Diego, CA). Written informed consent and parental permission were obtained in accordance with Institutional Review Board guidelines. Patient characteristics are given in **Table S1 **in File **S1**. Patients were treated according to a standard protocol with PEG-l-asparaginase, vincristine, and a glucocorticoid (prednisolone/prednisone or dexamethasone for children <10 or >10 years of age, respectively). The patients were hospitalized at the beginning of induction therapy (day 0) and released on day 8 of treatment. BM specimens were obtained on day 0 and at the end of induction therapy (day 29). PB specimens were collected on days 0, 8, and 29. Complete details of the induction therapy protocol are provided in **Table S12 **in File **S1**.

### Sample preparation for metabolomic analysis

A total of 50 samples were analyzed using MRS and GC-MS analytical platforms. Within 6 h of collection, the heparinized BM and PB specimens were centrifuged at 400 *g* for 20 min at 18°C. Aliquots of the supernatants (500 μL) were removed, immediately snap frozen in liquid nitrogen, and then stored at -80°C. The BM and PB biofluids were processed as previously described[29]. In brief, the frozen biofluids were thawed on ice and then deproteinized by ultrafiltration (Nanosep 3K OMEGA, Pall Corporation, MI) at 4°C. The filtrate polar fraction was prepared for MRS analysis as described in SI. Components remaining on the filter (mostly proteins and lipids) were recovered by washing with 1 mL 0.9% saline solution and prepared for analysis of the apolar fraction by GC-MS and MRS (details in SI).

### Preparation of polar fraction for MRS analysis

An aliquot of 160 μL of filtered biofluid was placed in a 3 mm MRS tube (Norell, Landisville, NJ, USA) containing 40 μL deuterated (3-(trimethylsilyl)-2,2',3,3'-tetradeuteropropionic acid (TMSP-d4, final concentration 0.5 mM; Cambridge Isotope Laboratories), 0.75% (w/v) sodium azide, phosphate buffer (final concentration 100 mM, pH 7.0) and 10% D_2_O (Cambridge Isotope Laboratories). Samples were analyzed immediately.

### Preparation of free fatty acid (FFA) extracts for GC-MS analysis

Three 25 μL aliquots of the recovered apolar fraction were prepared for GC-MS analysis of essential FFAs. Each aliquot was mixed with 475 μL saline, 500 μL methanol, 800 μL isooctane, 100 μL ethanol containing 25 ng of 98% myristic acid-d3 (Cambridge Isotope Laboratories), and 25 μL 1 N HCl. The solution was vortexed and the phases were separated by centrifugation. The upper isooctane layer was removed and dried with a vacuum evaporator for subsequent derivatization, as described [30]. In brief, the dried FFA fraction was redissolved in 25 μL 1% diisopropylethylamine in acetonitrile in capped glass tubes and derivatized by the addition of 25 μL 1% pentafluorobenzyl bromide (PFBB) in acetonitrile for 20 min at RT. The sample was dried and the residue was dissolved in 100 μL isooctane. For standard curves (1–500 ng), a mixture of arachidonic, palmitoleic, heptanoic, linolenic (all Sigma-Aldrich), palmitic, myristic, stearic, linoleic, oleic (all Fluka), and eicosadienoic (Cayman Chemical Co.) acids were PFBB-derivatized as described above. All solutions included 25 ng of myristic acid-d3. 

### Preparation of apolar fraction for MRS analysis

Recovered apolar metabolites (925 μL) in glass vials were extracted by the addition of 75 μL saline, 1 mL methanol, and 2 mL chloroform followed by vortexing for 30 s [31]. The emulsion was centrifuged at 5000 *g* for 10 min at 4°C and the layers were allowed to separate by standing for 10 min. The non-polar chloroform layer was removed and dried, and the residue was redissolved in CDCl_3_ containing 0.03% trimethylsilane. Samples were analyzed immediately.

### GC-MS analysis

GC-MS analysis was performed on a Shimadzu QP2010 Plus GC-MS (Shimadzu Corp, Kyoto, Japan) equipped with an autosampler. Samples of 1 μL derivatized FFAs in isooctane were injected in pulsed splitless injection mode onto 15 m × 0.25 mm × 0.25 μm SHRXI-5ms column (Shimadzu). The GC oven temperature was set to ramp from 150°C to 240°C at 10°C/min, from 240°C to 270°C at 40°C/min, and then to hold at 270°C for 1 min. The injector and transfer line were kept at 250°C and 280°C, respectively. Methane was used as the ionization gas with a source temperature of 150°C. Data were acquired in the selected ion monitoring (SIM) mode, monitoring the [M-H]- anions of FAs. Selected masses were arranged into eight SIM groups according to elution times. Calibration curves of saturated and unsaturated FFAs were generated by linear regression analysis of the individual lipid standards. For quantification of the remaining FFAs, the FFA standards with the closest chemical features were used.

### MRS experiments and data processing

MR spectra were acquired at 14.1 T (600 MHz) and 16.4 T (700 MHz) on Bruker Avance spectrometers (Bruker BioSpin Corp., Billerica, MA, USA) equipped with a TCI cryoprobe and autosampler at 30°C. Each sample was allowed to equilibrate for 10 min inside the probe before starting data acquisition. 

For the polar fraction, 1D ^1^H-MRS pulse sequence was implemented with excitation sculpting to suppress the water signal. 1D ^1^H-MRS spectra were acquired as specified by Chenomx NMR Suite (version 6.0; Chenomx Inc., Edmonton, Canada) for absolute metabolite quantification. For quantification of metabolites not present in the Chenomx library, an array of 1D MRS experiments was performed on representative samples under fully relaxed conditions (60 s recycle delay).

For the lipid fraction, Carr-Purcell-Meiboom-Gill (CPMG) ^1^H spectra were recorded using a spin-spin relaxation delay of 100 ms to facilitate detection of low molecular weight metabolites. For absolute quantification of lipid signals, nuclear Overhauser effect spectroscopy (NOESY) spectra were recorded with a mixing time of 10 ms. 

For all samples, 1D ^1^H-MRS spectra were acquired using at least 512 scans and 8 dummy scans, 32000 data points, and a spectral width of 6 kHz.

To facilitate metabolite identification, ^1^H-^13^C heteronuclear single-quantum coherence (HSQC) spectra were acquired on representative samples. For HSQC experiments, a total of 256 FIDs were recorded for each of 512 increments with a relaxation delay of 1.8 s. 

All the MRS datasets were processed using MetaboLab [32] in the MATLAB programming environment (MathWorks, Inc., Natick, MA). Post-processing of 1D MRS spectra for the multivariate analysis included scaling according to the probabilistic quotient method, alignment, exclusion of selected signals arising from solvents and TMSP, binning at 0.005 ppm, and application of a generalized log transformation. MRS resonances were assigned and the metabolites quantified using the Chenomx NMR Suite and other available libraries [22,30,33].

### Statistical analysis

Multivariate paired data analyses, mPCA and mPLS-DA, were performed on the entire MRS spectra using PLS-Toolbox (Version 6.5; Eigenvector Research, Manson, WA) in MATLAB. The absolute concentrations of selected metabolites are reported as mean values ± SEM. Statistical comparison of metabolite concentrations in different biofluids was performed using nonparametric WRST. The results were then FDR-corrected [34] to a significance level of 10%. Pearson’s correlation coefficients of metabolite concentrations were calculated and the highest correlations (r| > 0.75) were displayed in heat maps. P-values for the Pearson’s correlation coefficients were also calculated. Unsupervised hierarchical clustering analysis was performed using city-block distance and average linkage clustering methods on the Pearson’s correlation coefficients of all the identified metabolites. 

### Network analysis

The relevance network was obtained by calculating the mutual information and p-values of Pearson’s correlations among all pairs of metabolites with at least five nonzero values. Given the small sample size (10), we used a continuous method to estimate the mutual information (Spearman’s estimator [35]). As a criterion for relevance, we used an FDR-based cut-off (FDR <50%, corresponding to a cut-off in p-value of 0.063 for B0-P0 and 0.080 for B29-P29). Gaussian kernel smoothing restricted to positive values was used to generate smooth histograms, and the classification of the metabolites in the three groups (i-iii) was obtained from the Human Metabolome Database (http://www.hmdb.ca). The statistical analysis for significance (Mann-Whitney) was obtained using the Hypothesis Tests utilities of Mathematica (Wolfram Research, Inc.). The ARACNE network was calculated using the minet R/Bioconductor package [35] and plotted with Graphs and Networks utilities of Mathematica. Here, we also used a continuous variable estimator for the mutual information (Pearson’s estimator, default method in minet). Then, on each triplet of nodes (i,j,k), the edge corresponding to the smaller mutual information, for example (ij), was removed if its mutual information was below min{(ik),(jk)} [36]. The threshold for removing edges was set to zero, meaning that all triangular patterns have been removed from the graph. To further visualize regions of the networks with higher-than-average correlation, we removed edges corresponding to FDR <1% (calculated on all edges remaining after applying ARACNE). The resulting networks consist of the three connected components with more than three nodes. The three largest connected components in the B0-P0 network are characterized by the following properties: (a) 32 nodes; 33 edges; <R^2^> = 0.79, <p-value> = 0.001, <MI> = 0.84. (b) 8 nodes; 7 edges; <R^2^> = 0.79, <p-value> = 0.001, <MI> = 0.84. (c) 8 nodes; 7 edges; <R^2^> = 0.77, <p-value> = 0.001, <MI> = 0.74. The second connected component consists uniquely of type (i) edges. For the B29-P29 network, the three largest connected components are characterized by the following properties: (a) 19 nodes 19 edges; <R^2^> = 0.84, <p-value> = 0.0005, MI> = 0.95. (b) 6 nodes; 5 edges; <R^2^> = 0.76, <p-value> = 0.001, <MI> = 0.73. (c) 4 nodes; 3 edges; <R^2^> = 0.76, <p-value> = 0.001, <MI> = 0.72. The average < > is taken over the edges of the cluster and MI is the mutual information of the edges.

## Results

### Patient characteristics and sample collection

The study population was 10 patients (M:F ratio = 6:4) diagnosed with B-lineage ALL (**Table S1 **in File **S1**). The median age at diagnosis was 3 years (range, 1–14 years). BM and PB samples were obtained at diagnosis (day 0) and patients were started on l-asparaginase, vincristine, and glucocorticoid induction therapy on the same day. PB samples were taken on day 8, and both BM and PB samples were collected again on day 29, at the end of induction therapy. At the time of diagnosis, the median BM blast count was 86% (range, 45–93%) and the median PB blast count was 29.5% (range, 0–84%). All patients responded rapidly to therapy and the median PB blast count on day 8 was 0% (range, 0–12%). Only two patients had positive blast counts on day 8 (B005, 1% and B007, 12%), which corresponded to the minimal residual disease (MRD)-based risk group classification (0.1%≤ MRD<1%; additional information in SI). By day 29, all BM and PB blast counts were 0% except for one patient who had a BM blast count of 1%. All analytical samples were BM extracellular fluid (designated B0 and B29) or plasma (P0, P8, and P29). 

### Outline of metabolomic datasets and analytical approach

We used an untargeted approach to analyze metabolites in a range of metabolic pathways. Polar, whole lipid, and derivatized free fatty acid (FFA) fractions were prepared from a total of 50 biofluid samples (B0, B29, P0, P8, P29 for each of 10 patients) and analyzed by MRS (polar and apolar fractions) or GC-MS (FFAs). We detected and quantified a total of 102 metabolites: a summary of all results is shown in Table **1** and **Table S4 **in File **S2**. More detailed comparisons are shown in **Tables S5, S6, S7, S8, S9, S10, S11 **in File **S2**. Our analytical approach was designed to identify a metabolic profile that reflected cancer metabolism directly (B0), but comparisons with metabolites in the PB sample are useful to estimate the contribution of other factors affecting the cancer microenvironment in the same patient. For example, a comparison of P0 and B0 provides information about plasma metabolites before and after the blood enters the bone marrow, whereas a comparison of B0 and B29 provides information about metabolites in the BM niche in the presence and absence of cancer cells, with the caveat that metabolites in B29 samples might be affected by therapy.

**Table 1:** Metabolite concentrations in bone marrow and peripheral blood before and after induction therapy.

**Metabolites**	**B0 (μM)**	**P0 (μM)**	**B0-P0 (μM)**	**B29 (μM)**	**P29 (μM)**	**B29-P29 (μM)**
Lactate	3418.6	2378.42	1040.27	3648.98	4067.95	-418.96
Urea	5018.23	4464.38	553.85	8532.33	9175.15	-642.82
Glutamate	459.979	156.271	303.71	205.69	231.334	-25.64
Triacylglyceride	2299.07	2051.74	247.33	1488.55	1527.89	-39.34
Glycine	450.072	354.293	95.78	405.158	388.339	16.82
Glucose	5375.17	6066.73	-691.57	3764.65	4392.07	-627.42
Glycerol	450.072	354.293	-210.14	405.158	388.339	112.27
Cholesterol esters	3352.81	3545.79	-192.99	4085.96	4050.43	35.54
Glutamine	352.78	442.421	-89.64	380.068	372.481	7.59
3-Hydroxybutyrate	147.16	236.31	-89.15	41.72	62.46	-20.74

We used univariate analyses to assess changes in individual metabolites and multivariate statistical analysis to compare metabolomic profiles. Correlations among different metabolites in the 10 patients have also been reported because they might indicate compounds linked by metabolic pathways relevant to the cancer bone marrow microenvironment. The caveat here is that different cell types might contribute to the pathways.

### Metabolic differences between the BM and PB environments at diagnosis

**Figure 1:**
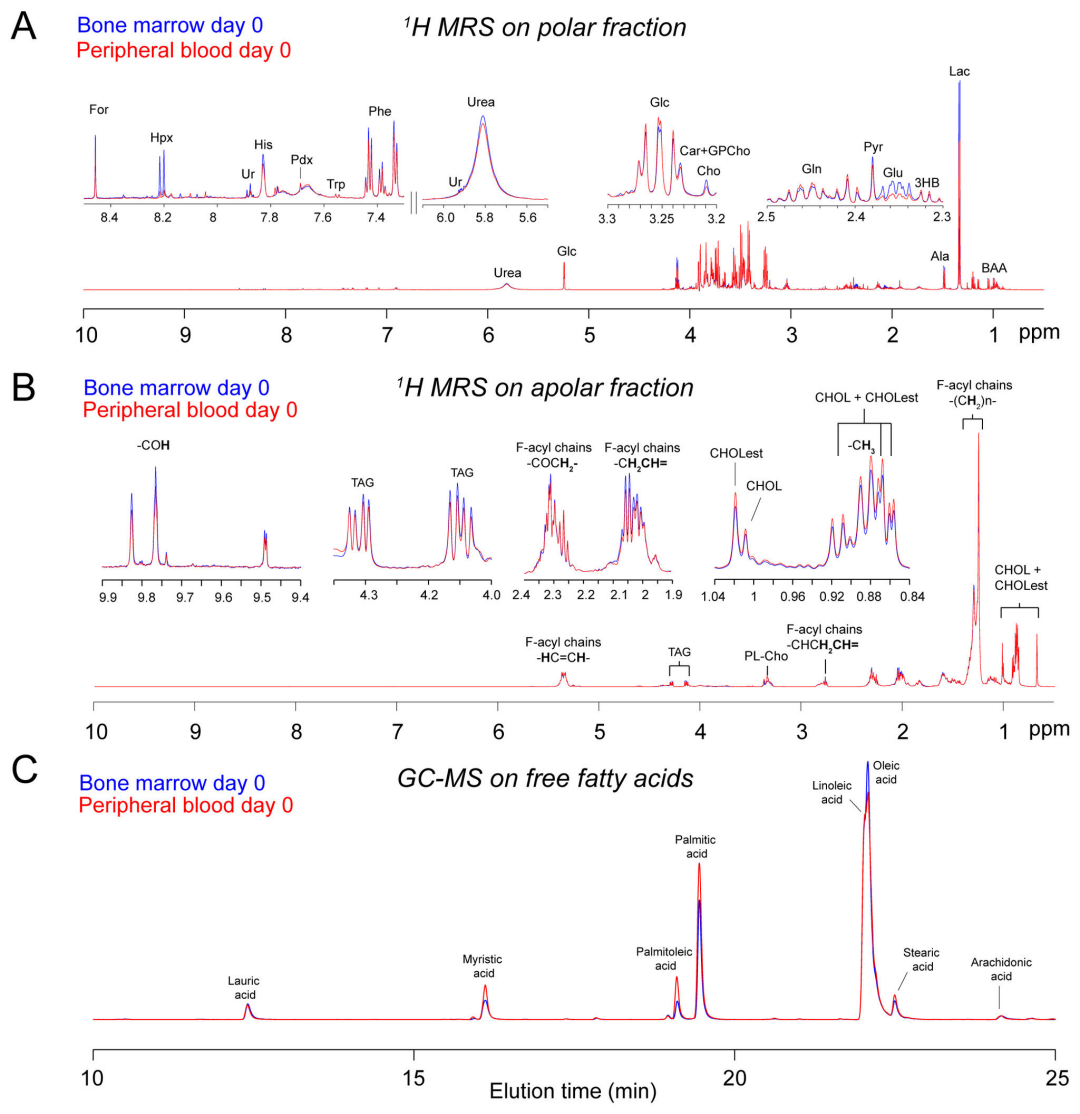
1H-MRS and GC-MS metabolic profiles of bone marrow and peripheral blood samples at the time of ALL diagnosis. Representative spectra of BM (blue line) and PB (red line) specimens. Spectra were acquired from (A) 1H-MRS analysis of filtered polar fractions, (B) 1H-MRS analysis of recovered whole lipid fractions, and (C) GC-MS analysis of FFA extracts. Metabolites with the greatest difference between BM and PB are labeled and include alanine (Ala), free cholesterol (CHOL), cholesterol esters (CHOLest), choline (Cho), formate (For), glucose (Glc), glutamate (Glu), glutamine (Gln), lactate (Lac), histidine (His), hypoxanthine (Hpx), palmitic acid, oleic acid, triacylglyceride (TAG), and uridine (Ur). Other abbreviations used are: (2HB), 2-hydroxybutyrate; (3HB), 3-hydroxybutyrate; (2Og), 2oxo-glutarate; (2Oic), 2oxo-isocaproate; (BAA), branched amino acids; (Car), carnitine; (Cho), choline; (CHOL), free cholesterol, (CHOLest), cholesterol esters; (For), formate; (Fum), fumarate; (Glyc), glycerol; (GPCho), glycero-3-phosphocholine; (Hpx), hypoxanthine; (Lac), lactate; (Niac), niacinamide; (Pglu), pyroglutamate; (Pyr), pyruvate; (Ur), uridine; (Pdx), pyridoxine; (TAG), triacylglyceride; (T-Chol), total cholesterol.

**Figure 2:**
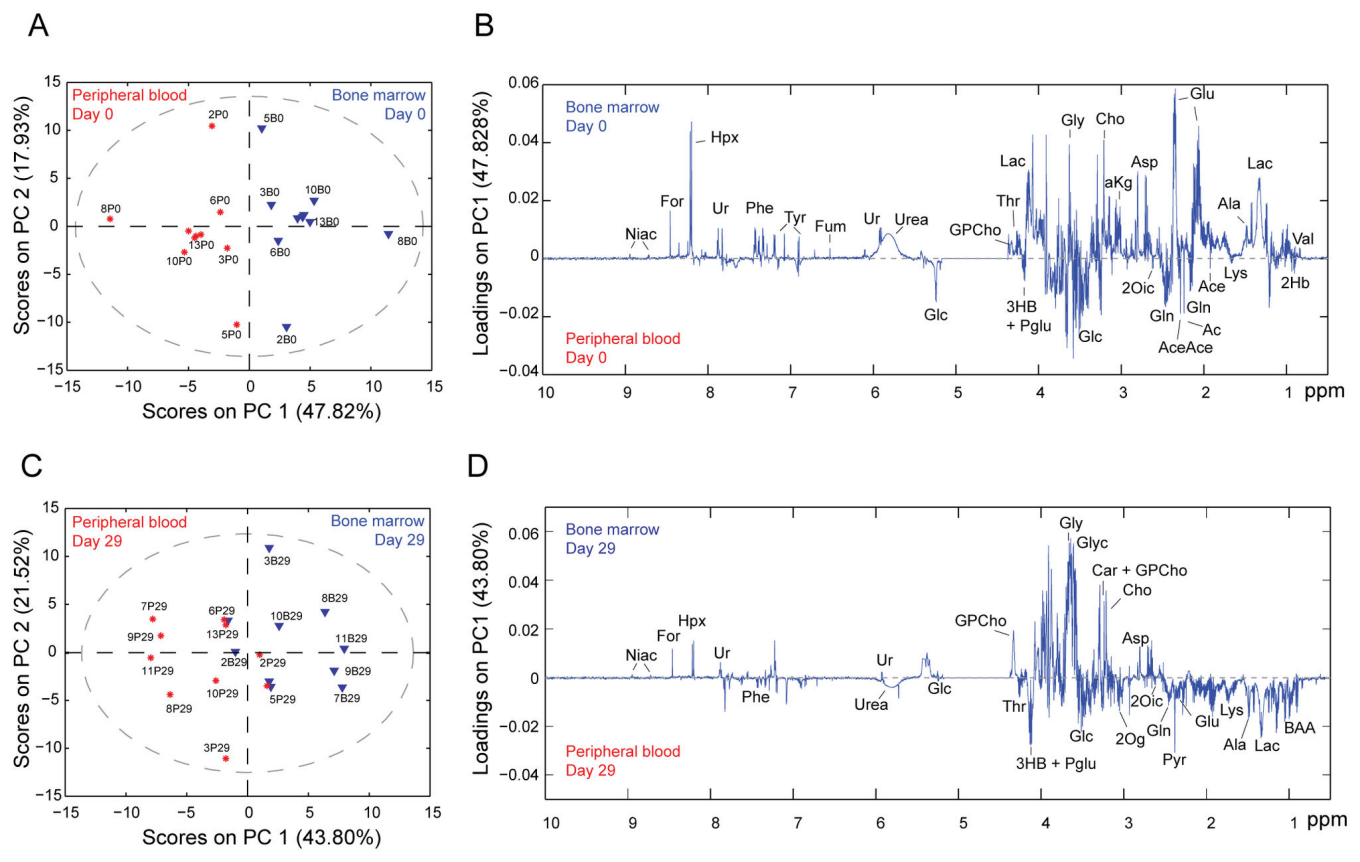
Untargeted multilevel principal component analysis of 1H-MRS spectra acquired on polar fractions of bone marrow and peripheral blood samples. (A, C) Scores plots obtained from mPCA performed on 1H MRS spectra of BM and PB samples collected at diagnosis (A, day 0) or after induction therapy (C, day 29). (B, D) Loadings plots for the first principal component depicts the most relevant discriminatory metabolites from BM (positive loadings) and PB (negative loadings) samples collected at diagnosis (B) and after induction therapy (D). Metabolites are defined in the Abbreviations section.

We first aimed to characterize the metabolome of BM and PB biofluids at the time of ALL diagnosis, when cancer cells almost completely fill the BM niche. The polar fraction of each biofluid was first characterized using one-dimensional (1D) MRS (Figure **1A**), and the spectra were analyzed by an untargeted multilevel principal component analysis (mPCA) to identify metabolites that differed between BM and PB (Figure **2A**). The scores plot showed an outstanding separation (47.82% of variance captured by the first principal component; Figure **2A**) and revealed important metabolic differences between BM and PB biofluids, as shown by the loadings plot (Figure **2B**). Notably, levels of valine, lactate, glutamate, aspartate, 2-oxoglutarate, choline, uridine, and hypoxanthine were elevated in the BM compared to the PB from the same patients, whereas levels of 2-hydroxybutyrate, 3-hydroxybutyrate, acetone, acetate, acetoacetate, lysine, glutamine, and glucose were reduced. 

**Figure 3:**
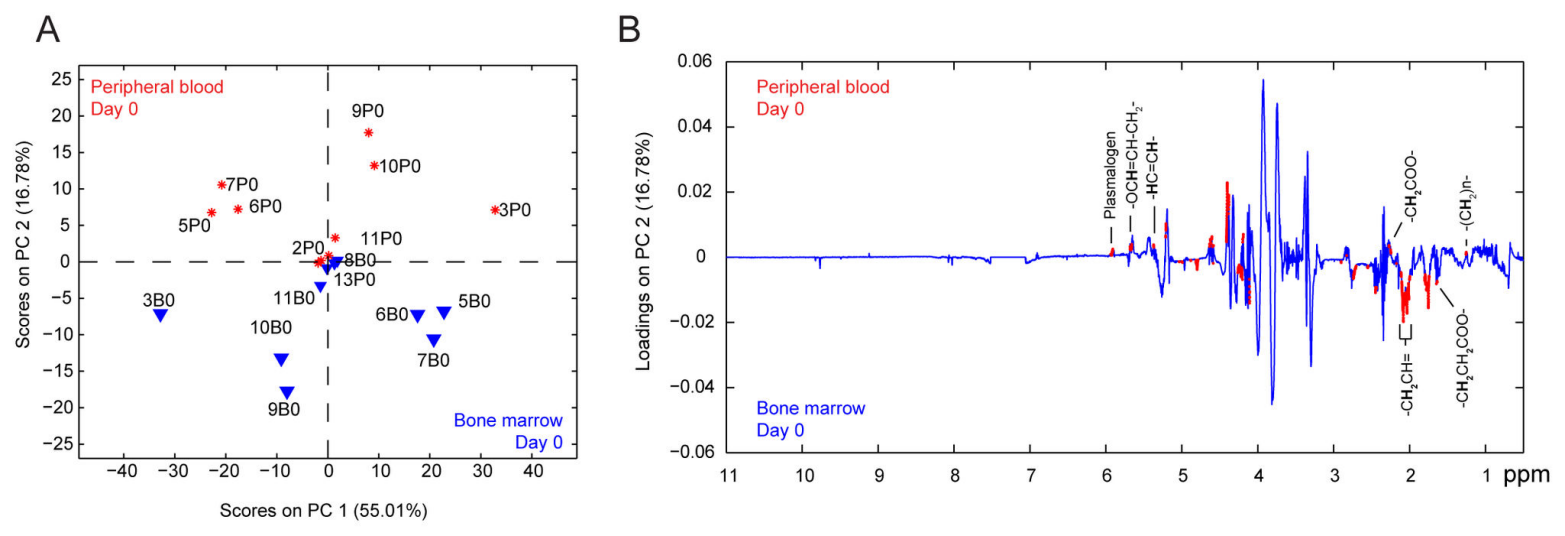
Untargeted multilevel principal component analysis performed on 1H-MRS spectra acquired on the whole lipid fraction of bone marrow and peripheral blood samples at the time of diagnosis. (A) mPCA scores plot shows a clear separation on the second principal component (PC2) between BM and PB specimens. (B) Loadings plot for the second principal component depicts the most relevant discriminatory functional groups from BM (negative loadings) and PB (positive loadings) collected at diagnosis. Red areas in (B) indicate significantly different regions of the MRS spectra according to a point-by-point nonparametric Wilcoxon Rank Sum Test (p < 0.05).

We next analyzed the whole lipid fractions of the biofluids by MRS (Figure **1B**) and conducted untargeted mPCA on the processed data. The mPCA score plot revealed a clear separation (16.78% PC2) between the BM and PB lipid fractions (Figure **3A**), albeit not as striking as that observed for the polar fraction of the same samples (Figure **2A**). To identify the functional groups that best discriminate between the BM and PB samples, we performed a point-by-point nonparametric Wilcoxon Rank Sum Test (WRST; p <0.05) analysis on the MRS spectra, and found that the most significant changes were mainly associated with FFAs, as highlighted on the loadings plot (Figure **3B**). Therefore, we isolated the FFA fraction from total lipids and performed a more in-depth analysis using GC-MS (Figure **1C**). 

**Figure 4:**
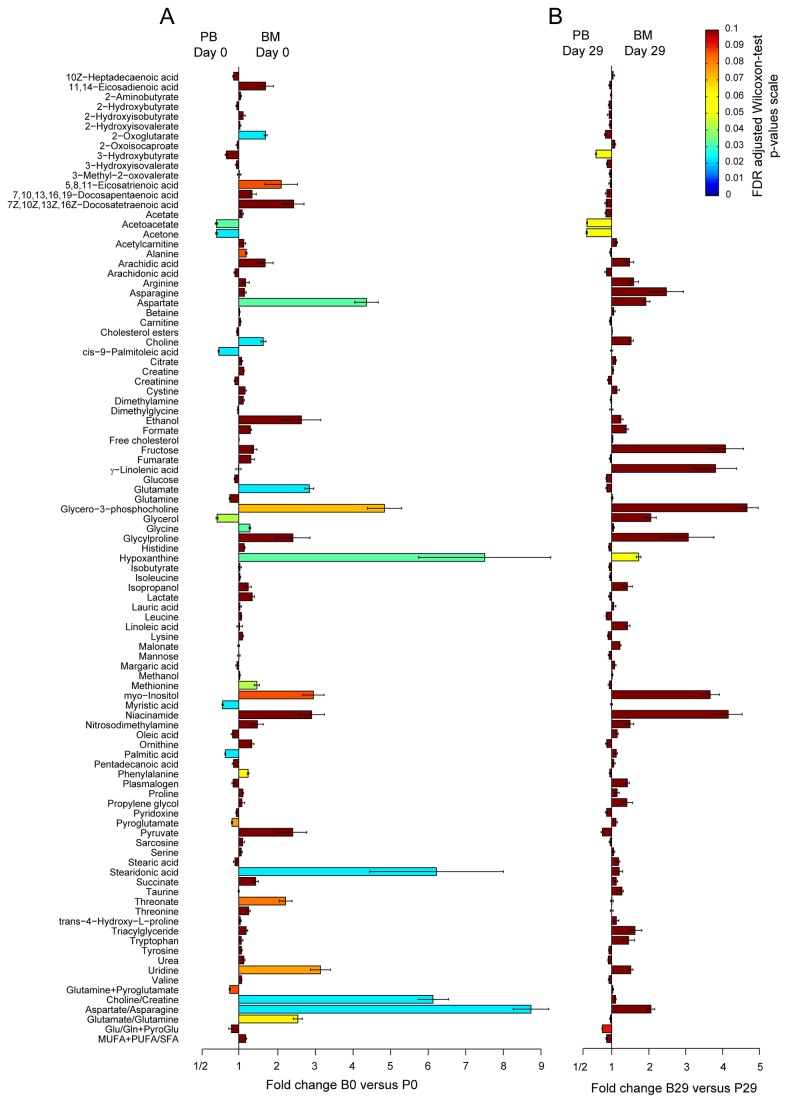
Targeted metabolic analysis of bone marrow and peripheral blood samples at diagnosis and after induction therapy. Shown are the mean fold differences in metabolite concentrations in BM and PB samples collected (A) at the time of diagnosis and (B) after induction therapy (mean ± SEM, n = 10 patients). Statistical significance was assessed based on absolute metabolite concentrations (SI, Table S1) using the nonparametric two-sided Wilcoxon Rank Sum Test (WRST) and p-values were corrected using false discovery rate (FDR). The bar plot is color coded according to p-values (pFDR < 10%). At day 0 (A), 22 metabolites were significantly different between BM and PB with pFDR <10%, 14 of which had pFDR <5%. In contrast, only 4 of 110 identified metabolites were found to be significantly different between BM and PB at day 29 (B). Both bar plots also show the differences in the ratios of glutamate to glutamine, aspartate to asparagine, choline to creatine, unsaturated to saturated fatty acids, and the sum of glutamine plus pyroglutamate.

In total, the polar, apolar, and FFA analyses identified 102 metabolites from the B0 and P0 samples of ALL patients (Figure **4A** **; Table S2 **in File **S1**). Selected metabolites that were not detected in all patients were omitted from the subsequent analysis (e.g., adipate and allantoin). Differences in the absolute metabolite concentrations in B0 and P0 were assessed for statistical significance using a nonparametric two-sided WRST test and corrected using the false discovery rate (FDR). We identified 27 metabolites with p-values <0.05; which was reduced to 22 with corrected pFDR<10% and 14 with pFDR<5% (Figure **4A**). The metabolites with higher significance (pFDR<5%) included 2-oxoglutarate, aspartate, choline, glutamate, glycine, hypoxanthine, and methionine, which were present at higher levels in BM than in PB, and acetoacetate, acetone, pyroglutamate, glycerol, myristic, *cis*-9-palmitoleic, and palmitic acids, which were present at higher levels in PB. Metabolites with lower significance (5%<pFDR<10%) were alanine, glycero-3-phosphocholine, myo-inositol, phenylalanine, threonate, and uridine, all of which were present at higher levels in BM.

We next created a correlation matrix between pairs of metabolites for each biofluid (**Tables S4 and S5 **in File **S2**), and the subsequent hierarchical clustering analysis (HCA) revealed that several metabolites were highly correlated (**Figures S2 and S3 **in File **S1**). In the BM (B0), we found 179 metabolite pairs having absolute Pearson’s correlation |r|>0.75 (p<0.01), 53 with |r|>0.85 (p<0.001), and 22 with |r|>0.93 (p<0.0001; **Table S4 **in File **S2**). In the PB (P0), we identified 151 correlations with |r|>0.75 (p<0.01), 44 with |r|>0.85 (p<0.001), and 18 with |r|>0.93 (p<0.0001; **Table S5 **in File **S2**). A strong correlation was found between the ketone bodies 3-hydroxybutyrate and acetoacetate in both microenvironments (in BM r=0.99, p=1.53×10^-08^; in PB r=0.97, p=4×10^-06^). To allow a simpler visualization of the differences between the two biofluids, we calculated the correlations as differences between the metabolite concentrations in BM and PB (**Table S6 **in File **S2**); the correlation values for all pairs of metabolites were then hierarchically classified using city-block distance and average linkage clustering methods (**Figure S4 **in File **S2**). In total, we found 161 pairwise correlations between metabolites having absolute Pearson’s correlation |r|>0.75 (p<0.01), 40 with |r|>0.85 (p<0.01), and 11 with |r|>0.93 (p<0.001). Remarkable correlations were found for several metabolite pairs that also clustered closely; for example, one cluster included fumarate with lactate (r=0.96, p=7×10^-06^), choline with tyrosine (r=0.96, p=1×10^-05^), and glutamate with choline (r=0.91, p=3×10^-04^), and a second cluster included *cis*-palmitoleic acid with acetone (r=0.97, p=4×10^-06^) *cis*-palmitoleic acid with myristic acid (r=0.94, p=4×10^-05^), and acetone with myristic acid (r=0.89, p=6×10^-04^). 

### Metabolic differences between the BM and PB environments after induction therapy

After 29 days of induction therapy, both BM and PB were largely tumor-free (**Table S1 **in File **S1**). The mPCA score plot for the polar metabolites (Figure **2C**) revealed good separation between the two biofluids (43.80% PC 1); however, the distribution of samples in the score plot did not show a clear separation, in contrast to the samples collected at day 0 (Figure **2A**). The loadings plot revealed that choline, glycerol-3-phosphocholine, glycerol, uridine, hypoxanthine, and formate were increased in BM relative to PB, whereas resonances from lactate, 2-hydroxybutyrate, 3-hydroxybutyrate, pyruvate, acetoacetate, acetone, and 2-oxoglutarate were reduced (Figure **2D**).

Multilevel chemometric analysis was next performed on MRS spectra acquired on the lipid fractions of biofluids collected at day 29. Using an unsupervised approach (mPCA), there was no clear separation between the groups (**Figure S5A **in File **S1**). The multilevel Partial Least Squares-Discriminant Analysis (mPLS-DA) model was then built on the same data using two classes and two latent variables (LVs), and 22.70% of variance was captured by LV1; **Figure S5B **in File **S1**). Validation using permutation tests determined that the model had significant predictive ability (Wilcoxon p ~0.01), thereby validating the need for a more detailed analysis of the data. Sensitivity and specificity values calculated for cross-validated mPLS-DA using Receiver Operating Characteristic (ROC) curve analysis were both 100%. However, a point-by-point nonparametric WRST (p<0.05) analysis on the MRS spectra (except plasmalogen) did not identify significant differences in the lipidomic profiles of the two biofluids after drug treatment (**Figure S5C **in File **S1**). 

Nonparametric WRST analysis of the polar, total lipid, and FFA fractions identified 18 metabolites with p-values <0.05, which was reduced to only 4 with pFDR<10% (3-hydroxybutyrate, acetoacetate, acetone, and hypoxanthine; Figure **3B**). Pearson’s correlation analyses for pairs of metabolites quantified in individual biofluids (**Tables S9 and S10 **in File **S2**) revealed particularly close affinity for pairs of amino acids and intermediate or byproducts of amino acid synthesis (**Figures S5, S6, S7 and S8 **in File **S1**). In addition, the heat map organized by HCA and obtained by subtracting the metabolite concentrations in PB from those in BM (**Figure S10 **in File **S1**) highlights the significant Pearson’s correlations of amino acids, including alanine and threonine (r=0.93, p=9×10^-05^), alanine and valine (r=0.96, p=8×10^-06^), isoleucine and methionine (r=0.94, p=3.9×10^-05^), betaine and dimethylglycine (r=0.98, p=1×10^-06^), isoleucine and phenylalanine (r=0.93, p=0.0001), and sarcosine and threonine (r=0.93, p=8×10^-05^; **Table S11 **in File **S2**). A comparison of bone marrow metabolites at day 0 and at day 29 is shown in **Figure S13 **in File **S1** and further comparisons are shown in **Figures S8 and S12 **in File **S1**.

### Longitudinal metabolite profiling of PB in response to chemotherapy

**Figure 5:**
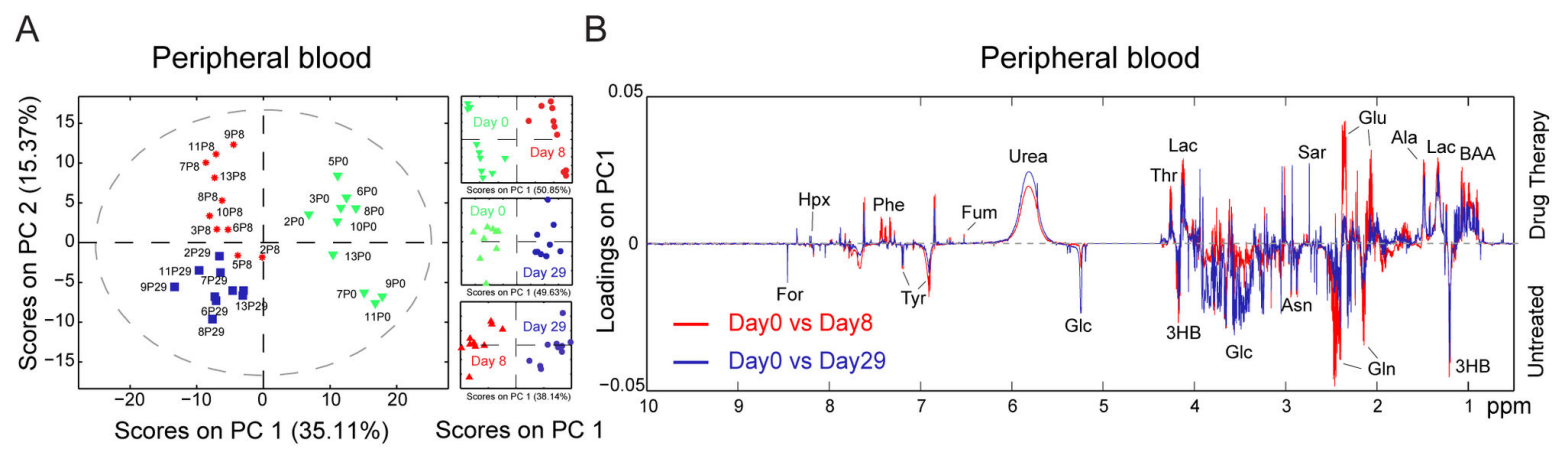
Multivariate analysis of peripheral blood polar fractions in response to drug therapy. Untargeted mPCA was performed on 1H-MRS spectra acquired on the polar fractions of PB (A, B). (A) mPCA scores plot shows a clear separation between PB samples collected on day 0 versus day 8 (49.63% on PC1), on day 0 versus day 29 (50.85% on PC1), and on day 8 versus day 29 (38.14% on PC1). (B) Loadings plot for the first principal components depicts the most relevant discriminatory metabolites for BM before therapy (negative loadings) and during or after therapy (positive loadings).

Blood samples were also collected on day 8 of therapy, which allowed us to further investigate the metabolic response of patients over the course of treatment by comparing the profiles of PB at days 0, 8, and 29. Notably, the median PB blast count at day 8 was 0% (range, 0–12%), indicating that all patients had a rapid response to therapy. Using mPCA on all of the MRS spectra acquired on the PB polar fractions, we obtained a very strong separation between PB from untreated and treated patients (**Figures S9A and S9B **in File **S1**). To maximize the information contained in each group and to identify the metabolites that are most affected by the chemotherapy regimen, we compared PB at day 0 versus day 8 (P0 vs P8; 49.63% on PC1), day 0 versus day 29 (P0 vs P29; 50.85% on PC1), and day 8 versus day 29 (P8 vs P29; 38.14% on PC1; Figures **5A and 5B**). For analysis of the lipid fraction, mPLS-DA models were built on the MRS data from the same samples and the significance of their predictivity was assessed by permutation testing (**Figure S10 **in File **S1**). The analyses indicated that drug therapy induced significant alterations in lipids, particularly after 8 days of treatment, as indicated by the resonances assigned to free cholesterol, cholesterol esters, triacylglyceride, plasmalogen, and saturated and unsaturated FFAs (**Figures S10A and S10B **in File **S1**). After 29 days, the differences in free cholesterol, cholesterol esters, and FFAs were still evident but were reduced in magnitude compared with the day 8 samples. 

The significance of the changes observed in the loadings plot was determined using WRST on the absolute metabolite concentrations. Comparing PB at days 0 and 8, 32 metabolites had p-values p<0.05, which was reduced to 25 metabolites with pFDR<10% and 16 with pFDR<5% (**Figure S11A **in File **S1**). Comparing PB at diagnosis and after induction therapy, 34 metabolites had p-values p<0.05, 30 of which had pFDR<10% and 22 had pFDR<5% (**Figure S11B **in File **S1**). Finally, comparing PB during (P8) and at the end (P29) of treatment, 24 metabolites had p-values p<0.05, which was reduced to 18 metabolites with pFDR <10% and 15 with pFDR<5% (**Figure S11C **in File **S1**). For the majority of the identified metabolites, the treatment-induced metabolic alterations observed at day 8 were maintained and remained significant at day 29. The most pronounced metabolic changes included alanine, asparagine, formate, fumarate, glutamate, lactate, sarcosine, and urea. However, there were also metabolites that showed different trends between day 0 to day 8 and day 8 to day 29. For example, glutamine, which would be expected to be strongly affected by l-asparaginase, a component of the drug treatment, showed a highly significant decrease after the first week of treatment (**Tables S4 and S5 **in File **S2**, **Figure S11 **in File **S1**; PB day 0 versus day 8; pFDR=0.0122), but increased by the end of therapy (PB day 8 versus day 29; pFDR=0.0133) almost to the same concentration measured at diagnosis (PB day 0 versus day 29; pFDR=0.1750). Untargeted mPLS-DA performed on ^1^H-MRS spectra acquired on the whole lipid fraction did also clearly separate bone marrow samples collected at day 0 and at day 29 (**Figure S12 **in File **S1**).

### A network analysis of the ALL cancer metabolome

**Figure 6:**
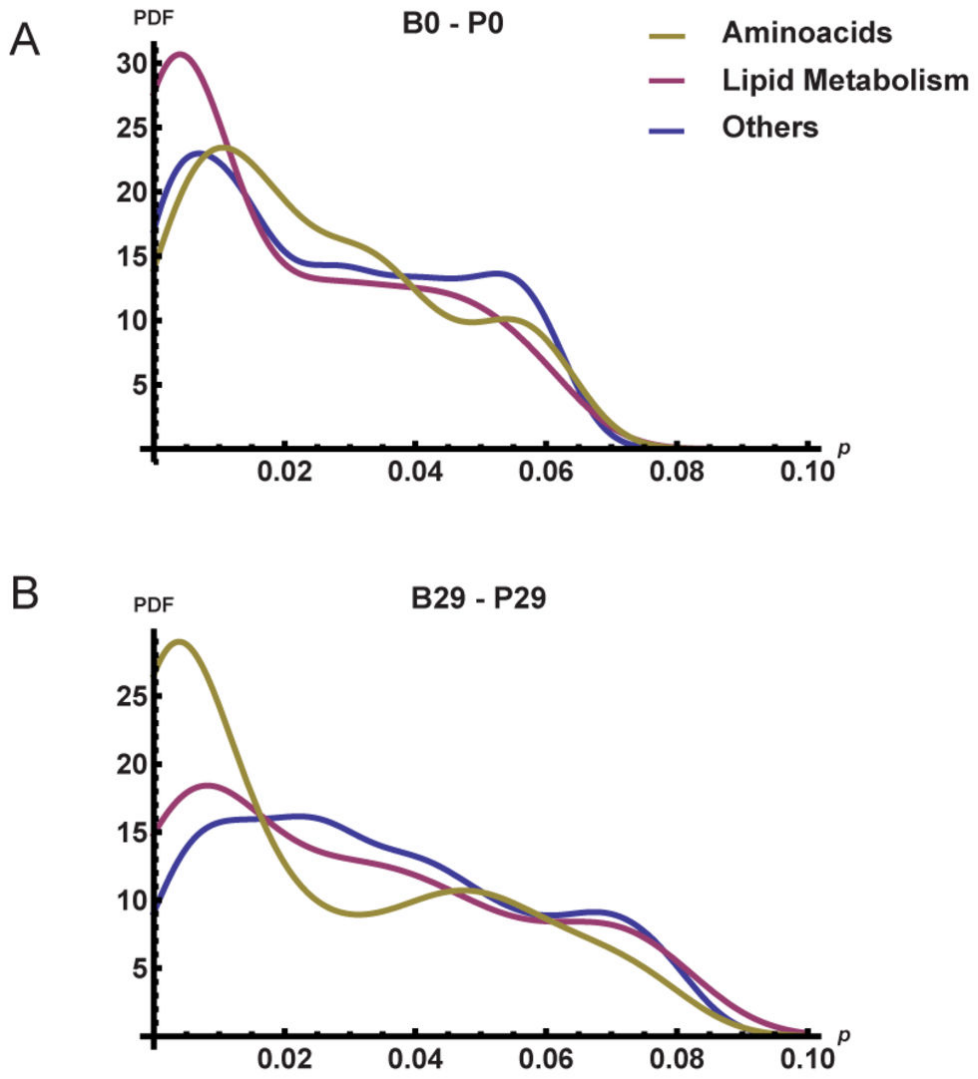
Statistical analysis of pairwise correlations of amino acids, lipid metabolites, and other metabolites. Plots show the probability distribution function (PDF) of the p-values of edges in a relevance network of metabolites with strong correlation in B0-P0 (A) and B29-P29 (B). Edges are classified in three groups (lipid metabolism, amino acids, and others). Note the presence of the high peak at small p-values in the lipid metabolite distribution in (A) and in the amino acid (including derivatives and analogues) distribution in (B), indicating enriched correlation among lipid metabolites at day 0 and amino acids at day 29, respectively.

Our quantitative MRS and GC-MS analysis indicated substantial changes in BM and PB metabolite levels between disease diagnosis and remission. We performed a network analysis of metabolites in the tumor microenvironment before (B0-P0) and after (B29-P29) induction therapy. Analysis of the datasets indicated that in the presence of cancer, correlation was strongest among metabolites involved in lipid metabolism (B0-P0; Figure **6A**), whereas correlation among amino acids (and analogues and derivatives) dominated after remission (B29-P29; Figure **6B**). We first used a relevance method to build a network of metabolites with pairwise correlation above a given threshold (see Methods). Edges were then classified into three groups: (i) edges between metabolites involved in lipid metabolism, (ii) edges between amino acids, derivatives, and analogues, and (iii) all other edges. We quantified the strength of the correlation by associating a p-value to each edge. Figures **6A and 6B** show the normalized distributions of the edge p-values (x-axis; *p*) for the B0-P0 and B29-P29 relevance networks, respectively. Note the presence of peaks at small p-values in the lipid metabolite distribution in Figure **6A** and in the amino acid distribution in Figure **6B**. A nonparametric Mann-Whitney test confirmed the enrichment of correlation among lipid metabolites (p<0.006) in B0-P0 and among amino acids (p<10^-9^) in B29‑P29. Using the same test, we also observed a significant difference (p=0.006) in the distributions of lipid metabolites in B0-P0 and in B29-P29, confirming an enrichment of correlation among lipid metabolites in the presence of cancer. 

**Figure 7:**
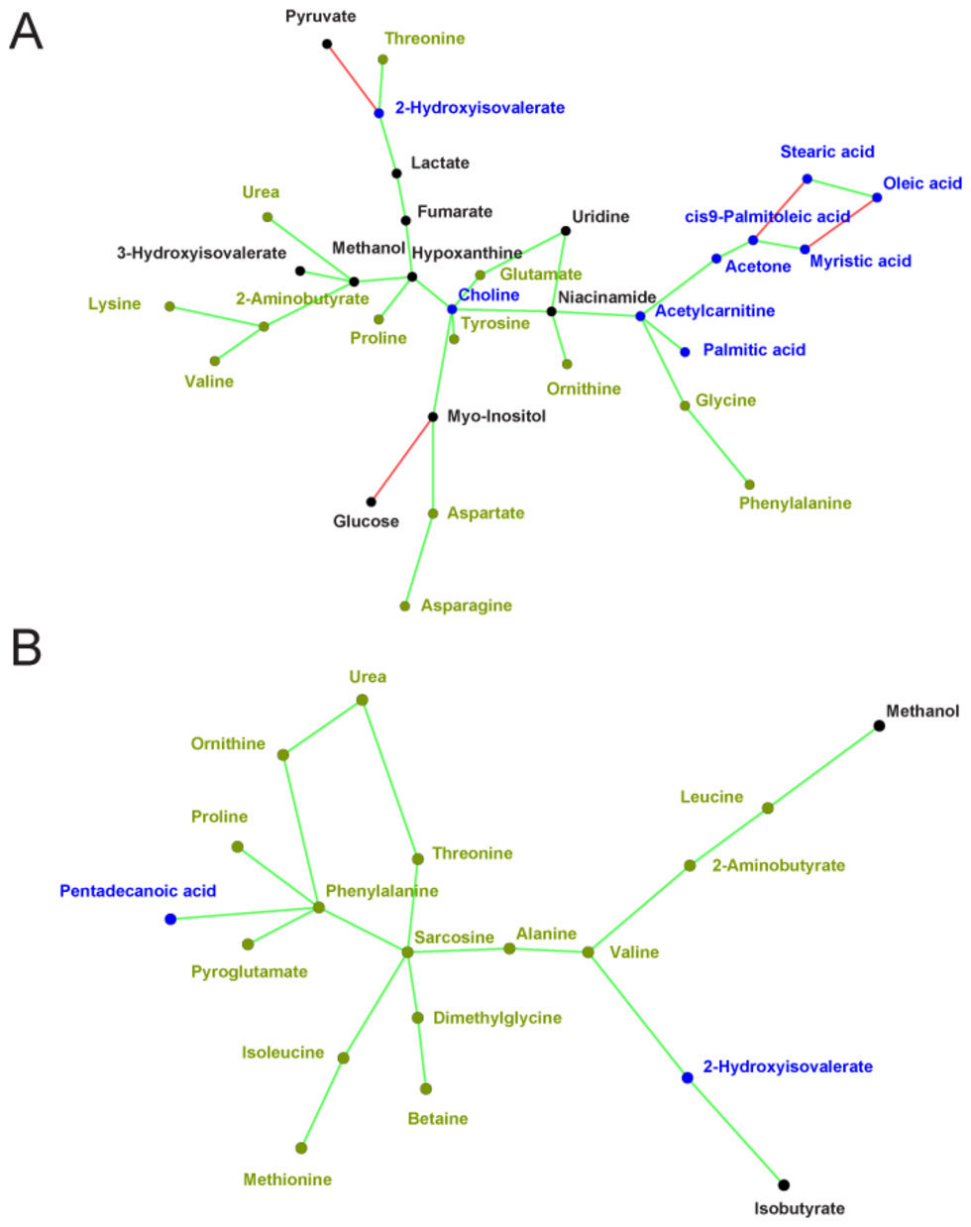
Network representation of correlations between metabolite pairs. Plots represent the largest connected component of the networks obtained with the ARACNE algorithm for B0-P0 (A) and B29-P29 (B). Blue nodes indicate metabolites relevant to lipid metabolism; green nodes indicate amino acids, including derivatives and analogues; red edges indicate anti-correlation; and light green edges indicate correlation. Shorter edges denote smaller p-values (higher R2). Note the presence of a community of lipid metabolites on the right side in (A) and the predominance of amino acids in (B).

To visualize the specific pathways associated with strong correlation, we used the ARACNE algorithm[36–38], which eliminates redundant edges derived from indirect correlations. Figures **7A and 7B** show the largest connected component of the B0-P0 and B29-P29 networks, respectively. The prevalence of interaction among lipid metabolites (indicated in blue) in the tumor environment at diagnosis is evident in the sub-network of FFAs on the right side of the figure, and two of the highest degree nodes, choline and acetylcarnitine, are key compounds in lipid metabolism (Figure **7A**). On the other hand, the switch in metabolism in the tumor-free environment is illustrated by the prevalence of interaction among amino acids, derivatives, and analogues at day 29 (indicated in green in Figure **7B**). Thus, the disappearance of lipid metabolism nodes on the B29-P29 network indicates that altered lipid metabolism is a specific signature of the cancer state in pediatric ALL. **Tables S2 and S3 **in File **S1** show more detailed pathway information for the metabolites of Figure **7**.

## Discussion

Pretreatment diagnosis of drug sensitivity would help therapeutic decisions and avoid unnecessary exposure of the patient to ineffective treatment and potentially life-threatening drug toxicity. One of the key challenges for personalized cancer therapy is to identify defined microenvironments that allow clinically relevant chemosensitivity testing of patients’ cells *in vitro*. Although some promising results have been reported[39], individualized chemosensitivity testing has never reached the level of acceptance of antibiotic sensitivity testing for bacterial pathogens[40]. However, achieving consistent clinical benefit for antibiotic testing required careful standardization of chemically defined media and testing conditions[41]. Our work might contribute to this important effort, especially in conjunction with measurements of cytokines and inorganic components of the cancer niche, which are now technically feasible[8],[42]. Current methods of tumor sensitivity testing rely on incompletely defined cell culture media that were developed decades ago simply to maximize cell growth for experimental convenience[43] but we now have suitable technologies to develop more realistic alternatives. Characterization of the tumor microenvironment may assist in the development of cell culture media that recapitulate *in vivo* conditions more closely and thus provide a more accurate prediction of the patient drug response, especially using primary cancer cells. **Figure S1 **in File **S1** shows a comparison of the patient bone marrow metabolic profile at day 0 with the commonly used cell culture medium RPMI. Our results show that, at the time of diagnosis, the BM ALL microenvironment has a metabolic signature clearly distinct from that of PB (22 metabolites with FDR<10%). This is most likely due to the very high accumulation of cancer cells in the BM at this stage of the disease. After therapy, the metabolic profiles of the BM niche and PB are very similar (4 metabolites with FDR<10%). 

Glutamate was significantly more abundant in BM than in PB before diagnosis, and was present at much higher levels than other amino acids. This difference cannot be attributed to an efflux of glutamate derived from glutamine. Our finding of a statistically significant correlation with the net glucose deficit is consistent with a study showing incorporation of glucose-derived carbons in glutamate molecules in an *in vitro* cancer model[44]. This partial oxidation of glucose is analogous to the well-known production of lactate in cancer cells[45,46]. We did indeed observe lower glucose levels and higher lactate levels in BM than in PB before therapy, consistent with many reports[10,47,48]. Metabolites present at significantly lower levels in BM before therapy might point to metabolic requirements of cancer cells that could be targeted for therapeutic purposes. As expected, concentrations of glucose and glutamine were much lower in BM than in PB at diagnosis, and the beneficial effect of asparaginase in ALL might be due, at least in part, to the deamination of circulating glutamine. It should be noted that a large number of the metabolites present at lower levels in BM before treatment are lipids and molecules involved in lipid metabolism. For example, lower concentrations of myristic, palmitic, and palmitoleic acids were found in the BM aspirates compared to the PB, suggesting a higher consumption of these FFAs by malignant lymphoblasts. Similarly, the significant accumulation of choline and glycero-3-phosphocholine in the BM reflect the abnormal metabolism of choline associated with oncogenesis[11]. We also report a trend towards increased consumption of cholesterol esters, which is small in percentage but large in terms of absolute consumption. The network analysis confirms the role of lipid metabolism in the cancer microenvironment of ALL patients. This analysis focused on pairwise correlation among metabolites rather than increases or decreases of single metabolites, and suggests the presence of a system-level cooperative behavior of lipid metabolites in the cancer state. A metabolic signature can be used to characterize the bone marrow cancer microenvironment, and, for example, could be used to monitor the microenvironment response to anticancer therapy. Correlation measures have also been used to infer the structure of biological networks that are relevant for a particular biological condition[49]. Similar network analyses based on correlation or mutual information in gene expression[37] have provided new insights into cancer biology and have been experimentally validated[37,38]. The findings of our network analysis are consistent with previously reported alterations of lipid metabolism in cancer[11,50]. A recent very large epidemiological study[51] reported an association between statin use and reduced cancer mortality, which confirmed data previous from *in vitro* studies[50] and suggested a possible relevant therapeutic intervention.

L-asparaginase is a component of chemotherapeutic regimens for pediatric ALL. Although it is generally thought that the sensitivity of leukemic lymphoblasts to this agent is due to their relatively low expression of asparagine synthetase[15–19], other studies suggest that glutamine depletion may be of therapeutic importance[52]. Asparaginase-induced depletion of circulating asparagine and glutamine might ultimately induce cell death by affecting both tumor energy metabolism and macromolecular biosynthesis[16,19]. We found that asparagine was entirely depleted in both BM and PB from day 8 to the end of induction therapy, consistent with previous reports[53,54]. In contrast, glutamine levels in PB were depleted during the first week of treatment, but recovered considerably by the end of therapy. It remains to be seen whether a more pronounced and prolonged depletion of glutamine might be beneficial in drug-resistant patients. 

Metabolic concentration of normal pediatric plasma metabolites levels exported from the human metabolome database (HMDB)[33] has been included in **Figure S14 **in File **S1**. From this data it appears that several metabolites are altered in ALL patients plasma, including most amino acids (but not the branched ones), carnitine, choline, creatine, creatinine, glycerol and palmitic acid. The same figure would suggest that some metabolites do in fact return towards more “normal” levels at the end of the induction therapy. However, due to the fact that patients are still experiencing the effect of therapy at the end induction, the return of plasma metabolites to normal levels is not necessarily to be expected.

## Limitations and Conclusions

This work has several limitations, some of which were, for technical or ethical reasons, unavoidable. One of the limitations is that we could not measure metabolites from individual cellular components of the bone marrow and peripheral blood. We also did not obtain bone marrow samples from normal children. Other limitations are due to clinical variability and are common to all clinical metabolomics studies, but are at least in part addressed by our experimental design, in which comparisons are made between samples collected in the same patient.

Other approaches to the study of cancer metabolism are also limited and are often less comprehensive. For example, in vitro studies cannot replicate the complexity of the in vivo microenvironment. It is therefore clear that to advance our understanding of cancer metabolism we should integrate information from different in vivo and in vitro studies. 

With these caveats, we conclude that the observed metabolic characteristics of the bone marrow cancer niche, including changes in lipid metabolism, might suggest new hypotheses for therapeutic targeting and optimization of existing leukemia therapies.

## Supporting Information

File S1 **Supplementary Results, Supplementary Methods, Supplementary Tables S1-S3 and S12, Supplementary Figures S1-S14.**
(DOCX)

File S2 **Supplementary Tables S4-S11.**
(XLSX)
