# Supplementary material for: Metabolomics of the Tumor Microenvironment in Pediatric Acute Lymphoblastic Leukemia
Source: PLoS One. 2013 Dec 13;8(12):e82859. doi: 10.1371/journal.pone.0082859 (PMC3862732; doi:10.1371/journal.pone.0082859)
Supplement: File S1 — Supplementary Results, Supplementary Methods, Supplementary Tables S1-S3 and S12, Supplementary Figures S1-S14. (DOCX) [file pone.0082859.s001.docx]

**Supplementary Information**

**Metabolomics of the tumor microenvironment in pediatric acute lymphoblastic leukemia**

Stefano Tiziani, Yunyi Kang, Ricky Harjanto, Joshua Axelrod, Carlo Piermarocchi, William Roberts, Giovanni Paternostro

**Results**

**Metabolomic differences between standard culture media and the ALL BM microenvironment**

One of the goals of metabolomics is to help define optimal *in vitro* conditions for drug sensitivity testing of tumors, particularly with patient-derived primary cancer cells. To analyze the differences between the primary tumor niche *in vivo* and the most common culture medium, we characterized the metabolome of RPMI/10% FBS, the most common *in vitro* culture media used for leukemic cell lines and primary cells, and BM extracellular fluid of ALL patients at the time of diagnosis (**Figure S1**).We found striking differences in the metabolic content of the samples, even though the analyses were performed with the same technique (MRS analysis).

**Metabolite profiling of BM in response to chemotherapy (B0 vs B29)**

We also investigated the effect of drug therapy on the metabolic response of patients by comparing BM at diagnosis and after 29 days of treatment. Using unsupervised mPCA on the MRS spectra acquired on the polar fractions, we obtained a very strong separation between BM from untreated and treated patients (42.73% on PC1; **Figure S8C and D**). Significant elevations were observed in 3-hydroxyisovalerate, creatine, pyruvate, glycerol, glycero‑3‑phosphocholine, sarcosine, and urea between day 0 and day 29, whereas 3-hydroxybutyrate, glucose, formate, and asparagine signals were significantly reduced. mPLS-DA models built on the data from the MRS lipid spectra were assessed for significance by permutation testing of their predictivity (**SI, Figure S11**). Significant treatment-induced changes were observed for free cholesterol, cholesterol esters, plasmalogen, and saturated and unsaturated FFAs. Thirty-five metabolites were identified with p-values p < 0.05, which was reduced to 30 with pFDR <10% and 26 with pFDR <5% (**SI, Figure S12**).

**Methods**

*Patient characteristics and sample collection.* Paired BM and PB specimens from 10 children diagnosed with B-ALL were collected at the Rady Children’s Hospital (San Diego, CA). Written informed consent and parental permission were obtained in accordance with Institutional Review Board guidelines. Patient characteristics are given in **Table 1**. Patients were treated according to a standard protocol with PEG-l-asparaginase, vincristine, and a glucocorticoid (prednisolone/prednisone or dexamethasone for children <10 or >10 years of age, respectively). The patients were hospitalized at the beginning of induction therapy (day 0) and released on day 8 of treatment. BM specimens were obtained on day 0 and at the end of induction therapy (day 29). PB specimens were collected on days 0, 8, and 29. Only two patients had positive blast counts on day 8 (B005, 1% and B007, 12%), which corresponded to the minimal residual disease (MRD)-based risk group classification (0.1% ≤ MRD < 1%). Median white blood cell counts at days 0, 8, and 29 were 7.5 × 10^3^/μL (range, 4.8–100 × 10^3^/μL), 1.2 × 10^3^/μL (range, 0.5–2.4 × 10^3^/μL), and 5.7 × 10^3^/μL (range, 1.9–10 × 10^3^/μL), respectively. Each BM sample was immunophenotyped (**Table 1**). Median percentages on day 0 were: CD10+ 99% (range, 54–100%), CD19+ 98% (range, 38–100%), and CD20+ 26.5% (range, 3–96%). The median percentages on day 29 were CD10+ 2% (range, 1–5%), CD19+ 8% (range, 1–28%), and CD20+ 8% (range, 2–28%), respectively.

*Sample preparation for flow cytometry.* BM specimens were collected in heparin tubes, diluted 1:1 (v/v) with 0.9% NaCl, and maintained at room temperature (RT) until analysis. Cells were stained using a stain/lyse technique. Briefly, BM cells were incubated with the immunophenotyping monoclonal antibodies described below, and red blood cells were then lysed in FACS Lysing Solution (BD Biosciences, San Jose, CA, USA). Cells were washed and resuspended in phosphate buffered saline for analysis.

*Immunophenotypic analysis at diagnosis.* Each BM specimen was stained with a panel of antibodies to confirm the leukemic immunophenotype. Cells were incubated with two combinations of monoclonal antibodies: CD20-FITC/CD10-PE/CD45-PerCP/CD19-APC and CD9-FITC/CD34-PE/CD45-PerCP/CD19-APC (all antibodies from BD Biosciences) and cells were analyzed using a FACSCanto flow cytometer (BD Biosciences). Cells were gated based on CD45 staining versus side scatter and a minimum of 30,000 events were collected.

*MRD analysis on follow-up specimen.* MRD analysis was performed on day 29 BM specimens. Cells were stained with the same monoclonal antibody combinations used for immunophenotyping. Cells were gated on the basis of CD19+ staining and 300,000 events were collected. Leukemic cells were identified by their abnormal marker expression and adequate distribution characteristics. The percentage of cells with an MRD phenotype was calculated as [(number of cells with abnormal phenotype) / (total number of cells acquired)] × 100.

*Pathway analysis.* For classification and pathway information of the metabolites in Figure 7A and 7B, HMDB [1] is the primary database used. Three key attributes of the metabolites are noted from the database and summarized in tables S2 and S3, which are class, subclass and pathways, along with the rank score of the two networks using the PageRank algorithm [2]. The rank score is computed by calculating the eigenvectors of the adjacency matrix in the PageRank algorithm and the calculation is written in Python. The connection between any two metabolites is considered as a bi-directional linkage. Only the main connected components of the two networks are counted into the algorithm to compute the rank score.

[1] Wishart DS, Jewison T, Guo AC, Wilson M, Knox C, et al., HMDB 3.0—The Human Metabolome Database in 2013. Nucleic Acids Res. 2013. Jan 1;41(D1):D801-7. Pubmed: 23161693

[2] Brin, S.; Page, L. (1998). "The anatomy of a large-scale hypertextual Web search engine". Computer Networks and ISDN Systems 30: 107–117. doi:10.1016/S0169-7552(98)00110-X. ISSN 0169-7552

**Supplementary Table**


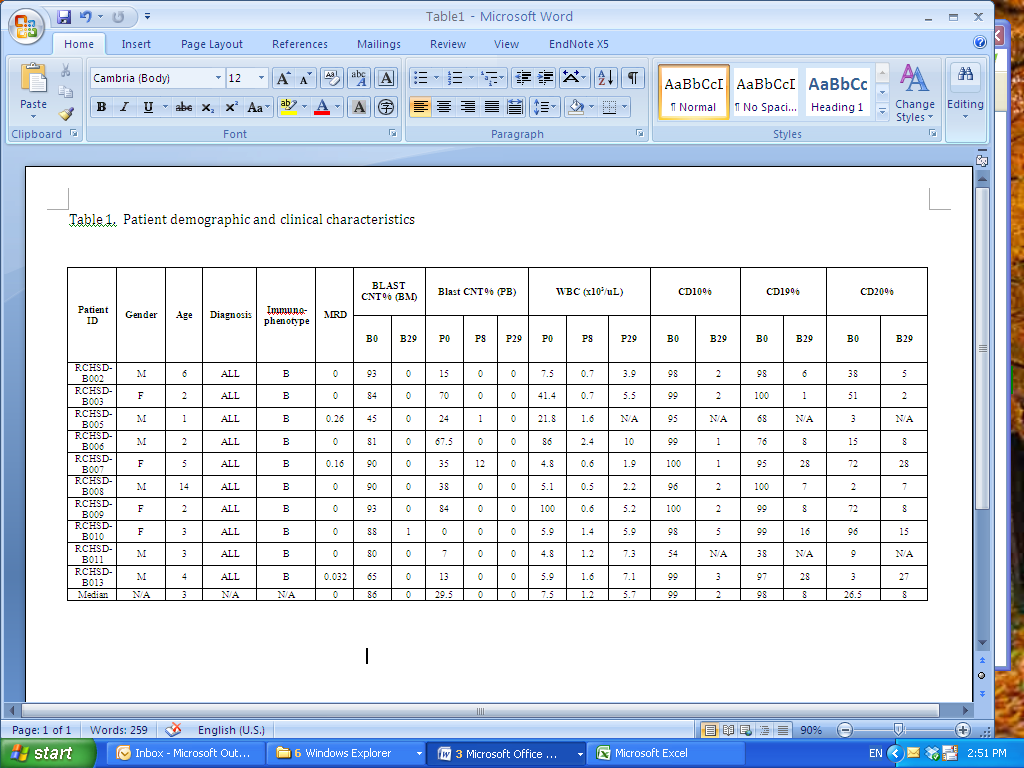


Table S1. Patient demographic and clinical characteristics.

MRD, mean residual disease; Blast CNT %, blast count; WBC, white blood cells.

| **metabolite** | **rank** | **class** | **subclass** | **pathways** |
| --- | --- | --- | --- | --- |
| Choline | 0.076 | Alcohols and Polyols | Cholines | Betaine Metabolism, Methionine Metabolism, Phospholipid Biosynthesis |
| Acetylcarnitine | 0.061 | Fatty Acid Esters | Acyl Carnitines | Beta Oxidation of Very Long Chain Fatty Acids, Oxidation of Branched Chain Fatty Acids |
| Hypoxanthine | 0.061 | Imidazopyrimidines | Purines and Purine Derivatives | Purine Metabolism |
| Methanol | 0.061 | Alcohols and Polyols | Primary Alcohols | No HMDB Pathway Information |
| Niacinamide | 0.061 | Pyridines and Derivatives | N/A | Nicotinate and Nicotinamide Metabolism |
| 2-Aminobutyrate | 0.045 | Amino Acids and Derivatives | Alpha Amino Acids and Derivatives | No HMDB Pathway Information |
| 2-Hydroxyisovalerate | 0.045 | Fatty Acids and Conjugates | Branched Fatty Acids | No HMDB Pathway Information |
| cis-9-Palmitoleic acid | 0.045 | Fatty Acids and Conjugates | Unsaturated Fatty Acids | No HMDB Pathway Information |
| Myo-Inositol | 0.045 | Cyclic Alcohols and Derivatives | Cyclitols and Derivatives | Galactose Metabolism, Inositol Metabolism, Inositol Phosphate Metabolism, Phosphatidylinositol Phosphate Metabolism |
| Acetone | 0.030 | Carbonyl Compounds | Ketones | Ketone Body Metabolism |
| Aspartate | 0.030 | Amino Acids and Derivatives | Alpha Amino Acids and Derivatives | Ammonia Recycling, Arginine and Proline Metabolism, Aspartate Metabolism |
| Fumarate | 0.030 | Fatty Acids and Conjugates | Unsaturated Fatty Acids | Arginine and Proline Metabolism, Aspartate Metabolism, Citric Acid Cycle, Mitochondrial Electron Transport Chain, Phenylalanine and Tyrosine Metabolism, Tyrosine Metabolism, Urea Cycle |
| Glutamate | 0.030 | Amino Acids and Derivatives | Alpha Amino Acids and Derivatives | Alanine Metabolism, Amino Sugar Metabolism, Ammonia Recycling, Arginine and Proline Metabolism, Cysteine Metabolism, Folate Metabolism |
| Glycine | 0.030 | Amino Acids and Derivatives | Alpha Amino Acids and Derivatives | Alanine Metabolism, Ammonia Recycling, Bile Acid Biosynthesis, Carnitine Synthesis, Glutathione Metabolism, Glycine and Serine Metabolism, Methionine Metabolism, Porphyrin Metabolism |
| Lactate | 0.030 | Hydroxy Acids and Derivatives | Alpha Hydroxy Acids and Derivatives | Gluconeogenesis, Pyruvate Metabolism |
| Myristic acid | 0.030 | Fatty Acids and Conjugates | Straight Chain Fatty Acids | Fatty Acid Biosynthesis |
| Oleic acid | 0.030 | Fatty Acids and Conjugates | Unsaturated Fatty Acids | No HMDB Pathway Information |
| Stearic acid | 0.030 | Fatty Acids and Conjugates | Straight Chain Fatty Acids | Plasmalogen Synthesis |
| Uridine | 0.030 | Pyrimidine Nucleosides and Analogues | N/A | Pyrimidine Metabolism |
| 3-Hydroxyisovalerate | 0.015 | Hydroxy Acids and Derivatives | Beta Hydroxy Acids and Derivatives | No HMDB Pathway Information |
| Asparagine | 0.015 | Amino Acids and Derivatives | Alpha Amino Acids and Derivatives | Ammonia Recycling, Aspartate Metabolism, Transcription/Translation |
| Glucose | 0.015 | Monosaccharides | Hexoses | Galactose Metabolism, Gluconeogenesis |
| Lysine | 0.015 | Amino Acids and Derivatives | Alpha Amino Acids and Derivatives | Biotin Metabolism, Carnitine Synthesis, Lysine Degradation, Transcription/Translation |
| Ornithine | 0.015 | Amino Acids and Derivatives | Alpha Amino Acids and Derivatives | Arginine and Proline Metabolism, Glycine and Serine Metabolism, Spermidine and Spermine Biosynthesis, Urea Cycle |
| Palmitic acid | 0.015 | Fatty Acids and Conjugates | Straight Chain Fatty Acids | Fatty Acid Biosynthesis, Fatty Acid Elongation In Mitochondria, Fatty acid Metabolism, Glycerolipid Metabolism |
| Phenylalanine | 0.015 | Amino Acids and Derivatives | Alpha Amino Acids and Derivatives | Phenylalanine and Tyrosine Metabolism, Transcription/Translation |
| Proline | 0.015 | Amino Acids and Derivatives | Alpha Amino Acids and Derivatives | Arginine and Proline Metabolism, Transcription/Translation |
| Pyruvate | 0.015 | Keto-Acids and Derivatives | Alpha Keto-Acids and Derivatives | Alanine Metabolism, Amino Sugar Metabolism, Ammonia Recycling, Citric Acid Cycle, Cysteine Metabolism, Gluconeogenesis |
| Threonine | 0.015 | Amino Acids and Derivatives | Alpha Amino Acids and Derivatives | Glycine and Serine Metabolism |
| Tyrosine | 0.015 | Amino Acids and Derivatives | Alpha Amino Acids and Derivatives | Catecholamine Biosynthesis, Phenylalanine and Tyrosine Metabolism, Transcription/Translation, Tyrosine Metabolism |
| Urea | 0.015 | Ureas | N/A | Arginine and Proline Metabolism, Urea Cycle |
| Valine | 0.015 | Amino Acids and Derivatives | Alpha Amino Acids and Derivatives | Propanoate Metabolism, Transcription/Translation |

Table S2. Metabolites classification and pathways from HMDB and rank score of metabolites in Figure 7A.

| **metabolite** | **rank** | **class** | **subclass** | **pathways** |
| --- | --- | --- | --- | --- |
| Phenylalanine | 0.132 | Amino Acids and Derivatives | Alpha Amino Acids and Derivatives | Phenylalanine and Tyrosine Metabolism, Transcription/Translation |
| Sarcosine | 0.132 | Amino Acids and Derivatives | Alpha Amino Acids and Derivatives | Glycine and Serine Metabolism |
| Valine | 0.079 | Amino Acids and Derivatives | Alpha Amino Acids and Derivatives | Propanoate Metabolism, Transcription/Translation |
| 2-Aminobutyrate | 0.053 | Amino Acids and Derivatives | Alpha Amino Acids and Derivatives | No HMDB Pathway Information |
| 2-Hydroxyisovalerate | 0.053 | Fatty Acids and Conjugates | Branched Fatty Acids | No HMDB Pathway Information |
| Alanine | 0.053 | Amino Acids and Derivatives | Alpha Amino Acids and Derivatives | Alanine Metabolism |
| Dimethylglycine | 0.053 | Amino Acids and Derivatives | Alpha Amino Acids and Derivatives | Betaine Metabolism, Glycine and Serine Metabolism, Methionine Metabolism |
| Isoleucine | 0.053 | Amino Acids and Derivatives | Alpha Amino Acids and Derivatives | Transcription/Translation |
| Leucine | 0.053 | Amino Acids and Derivatives | Alpha Amino Acids and Derivatives | Transcription/Translation |
| Ornithine | 0.053 | Amino Acids and Derivatives | Alpha Amino Acids and Derivatives | Arginine and Proline Metabolism, Glycine and Serine Metabolism, Spermidine and Spermine Biosynthesis, Urea Cycle |
| Threonine | 0.053 | Amino Acids and Derivatives | Alpha Amino Acids and Derivatives | Glycine and Serine Metabolism |
| Urea | 0.053 | Ureas | N/A | Arginine and Proline Metabolism, Urea Cycle |
| Betaine | 0.026 | Amino Acids and Derivatives | Alpha Amino Acids and Derivatives | Betaine Metabolism, Glycine and Serine Metabolism, Methionine Metabolism |
| Isobutyrate | 0.026 | Carboxylic Acids and Derivatives | Carboxylic Acid Derivatives | No HMDB Pathway Information |
| Methanol | 0.026 | Alcohols and Polyols | Primary Alcohols | No HMDB Pathway Information |
| Methionine | 0.026 | Amino Acids and Derivatives | Alpha Amino Acids and Derivatives | Betaine Metabolism, Glycine and Serine Metabolism, Methionine Metabolism, Spermidine and Spermine Biosynthesis, Transcription/Translation |
| Pentadecanoic acid | 0.026 | Fatty Acids and Conjugates | Straight Chain Fatty Acids | No HMDB Pathway Information |
| Proline | 0.026 | Amino Acids and Derivatives | Alpha Amino Acids and Derivatives | Arginine and Proline Metabolism, Transcription/Translation |
| Pyroglutamate | 0.026 | Pyrrolidines | Pyrrolidine Carboxylic Acids and Derivatives | Glutathione Metabolism |

TableS3. Metabolites classification and pathways from HMDB and rank score of metabolites in Figure 7B

| **Drug** | **Route** | **Dosage** | **Days** |
| --- | --- | --- | --- |
| Intrathecal Cytarabine (IT ARAC) | IT | Age (yrs) Dose  1-1.99 30 mg  2-2.99 50 mg  >= 3 70 mg | Given at time of diagnostic lumbar puncture OR Day1 |
| Vincristine (VCR) | IV push over 1 minute | 1.5 mg/m^2^/dose | Days 1, 8, 15, 22 |
| Dexamethasone (DEX) | PO  (may give IV) | 3 mg/m^2^/dose BID | Days 1-28 |
| PEG-asparaginase  (PEG-ASP) | IM | 2500 International units/m^2^X 1 dose | Day 4 or 5 or 6 |
| Intrathecal Methotrexate  (IT MTX) | IT | Age (yrs) Dose  1-1.99 8 mg  2-2.99 10 mg  3-8.99 12 mg  >=9 15mg | Days 8, 29 |

**Table S12. Drug and Dosage schedules of induction therapy** IT (intrathecal), PO (per os, by mouth), IM (intramuscular), IV (intravenous), BID (twice a day).

**Supplementary Figures**


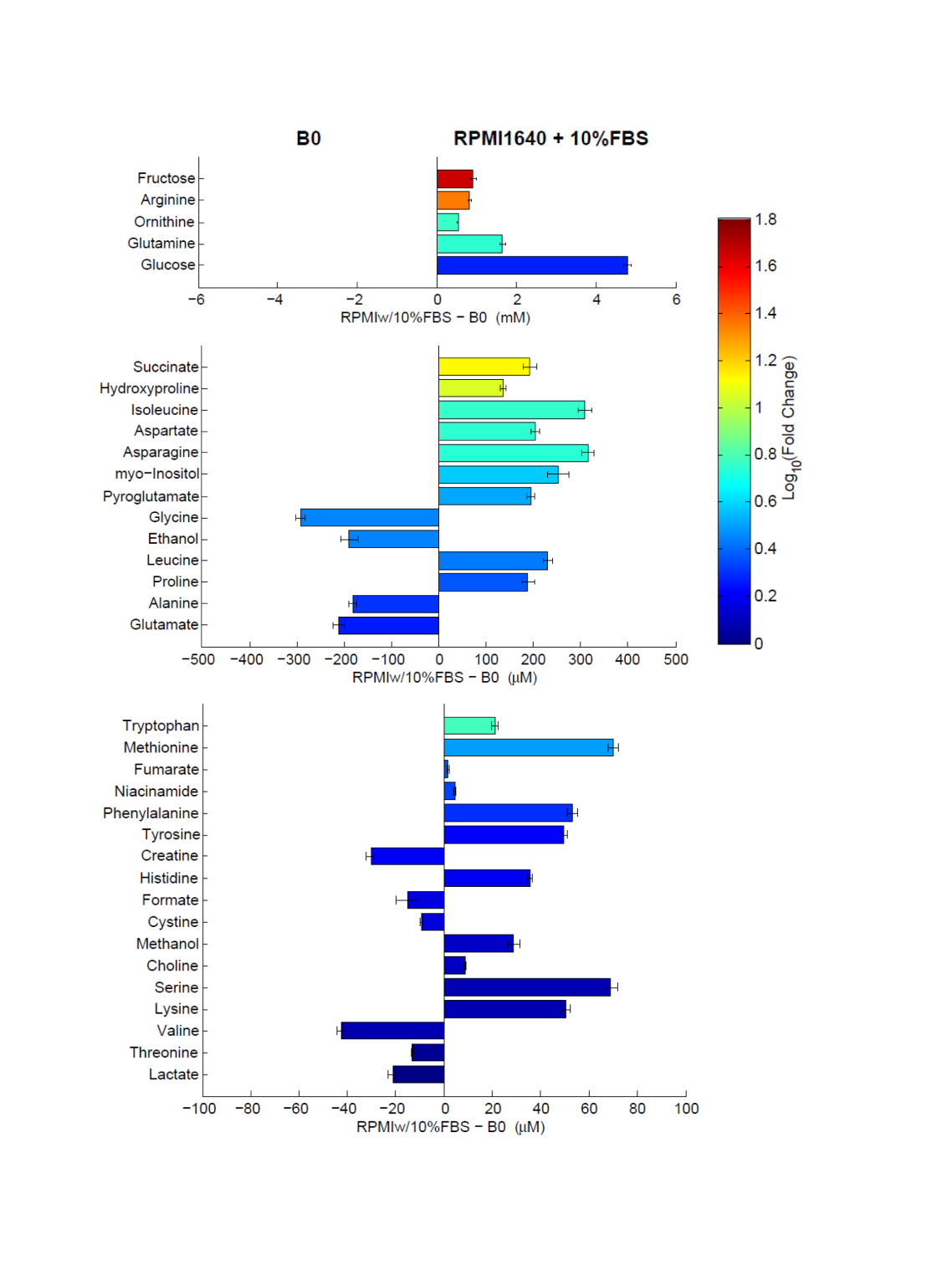


**Figure S1. Targeted metabolic analysis of patient bone marrow and standard culture medium.**

Shown are the fold differences (mean ± SEM) in metabolite concentrations in BM (day 0, n = 10 patients) and RPMI 1640/10% FBS.


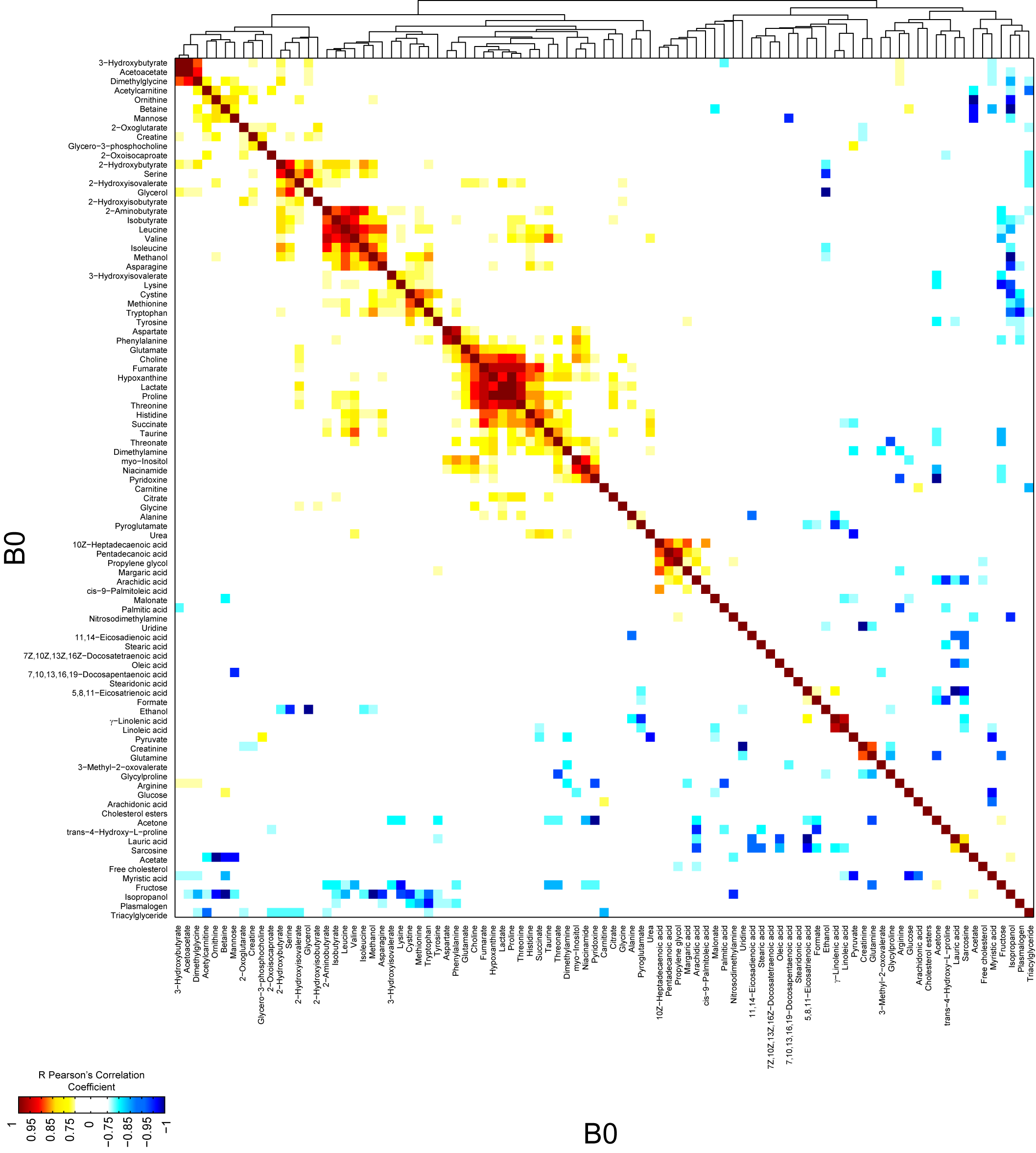


**Figure S2. Heat map of correlations between metabolites in bone marrow** **collected from ALL patients at diagnosis.**

Hierarchical clustering analysis of Pearson’s coefficients calculated from the absolute metabolite concentrations (**Table S1**) in BM samples collected on day 0. Only Pearson’s correlation coefficients r > 0.75 or r < ‑0.75 are shown. Pairs of metabolites are hierarchically classified using city-block distance and average linkage clustering methods. Individual correlation coefficients and p-values can be found in **Table S2**.


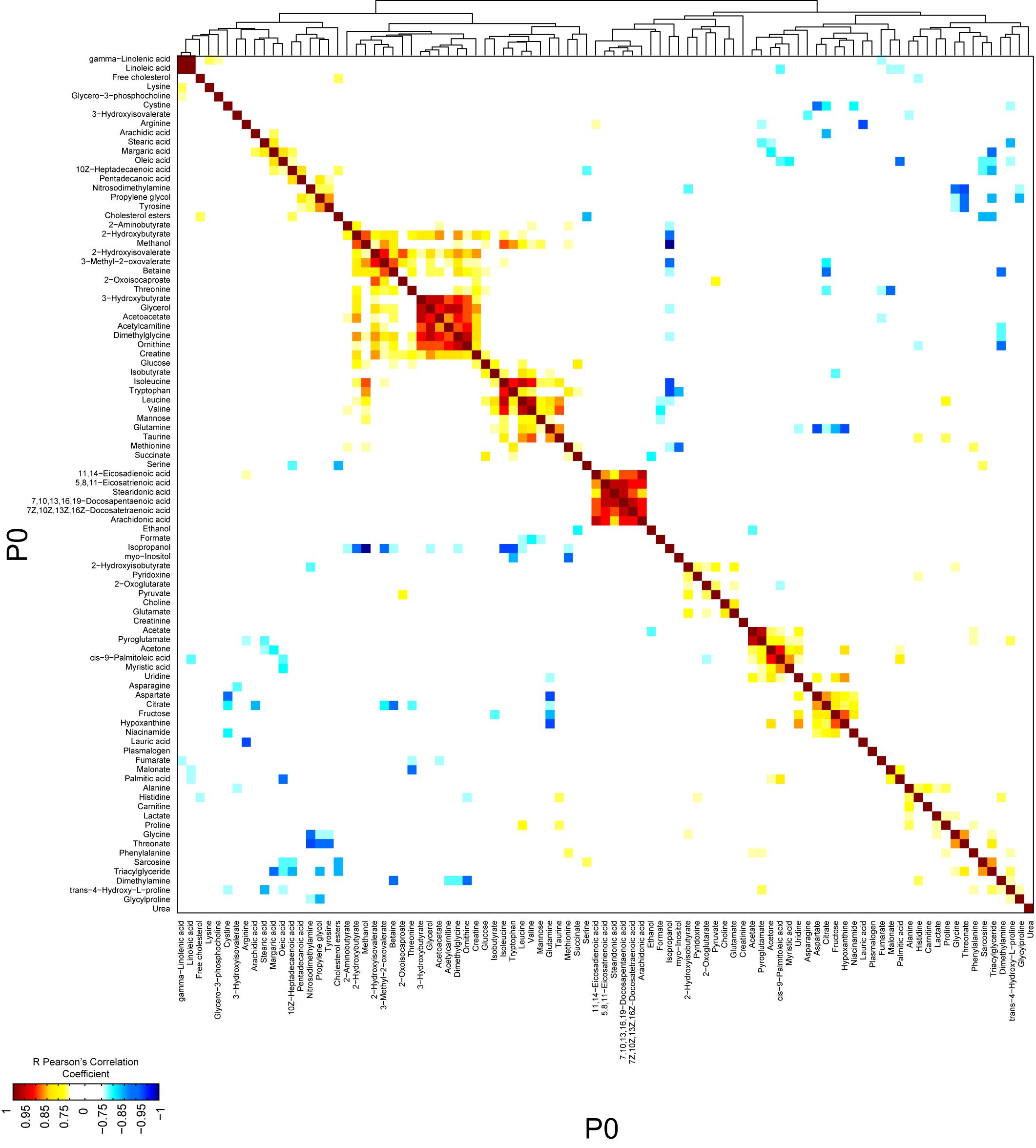


**Figure S3. Heat map of correlations between metabolites in peripheral blood samples collected from ALL patients at diagnosis.**

Hierarchical clustering analysis of Pearson’s coefficients calculated from the absolute metabolite concentrations (**Table S1**) in PB samples collected on day 0. Only Pearson’s correlation coefficients r > 0.75 or r < ‑0.75 are shown. Pairs of metabolites are hierarchically classified using city-block distance and average linkage clustering methods. Individual correlation coefficients and p-values can be found in **Table S3**.


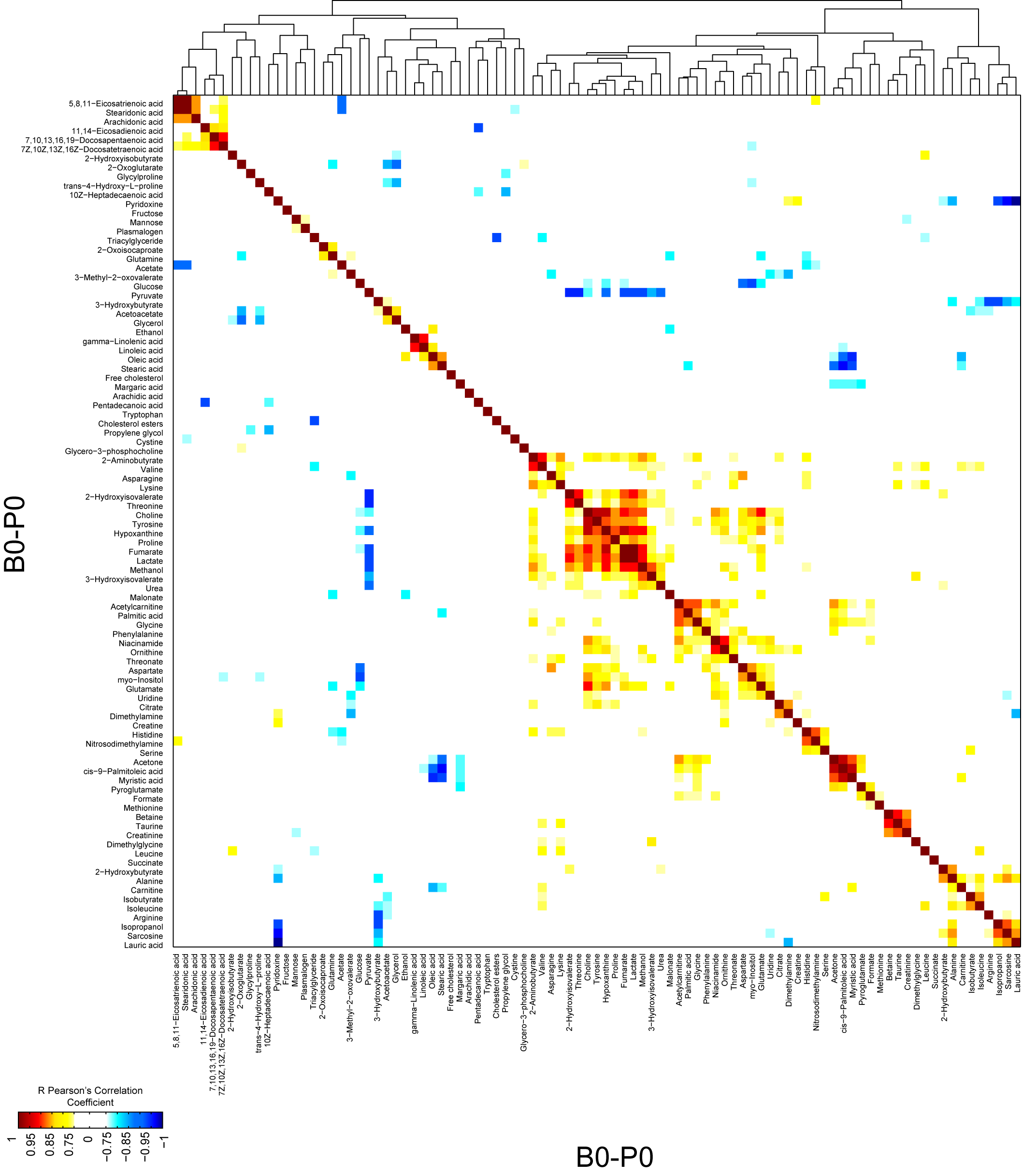


**Figure S4. Heat map of correlations between metabolic differences in bone marrow and peripheral blood samples collected at diagnosis.**

Hierarchical clustering analysis of Pearson’s coefficients calculated from the difference in absolute metabolite concentrations in BM and PB samples (B0-P0) collected on day 0. Only Pearson’s correlation coefficients r > 0.75 or r < ‑0.75 are shown. Pairs of metabolites are hierarchically classified using city-block distance and average linkage clustering methods. Individual correlation coefficients and p-values can be found in **Table S4**.


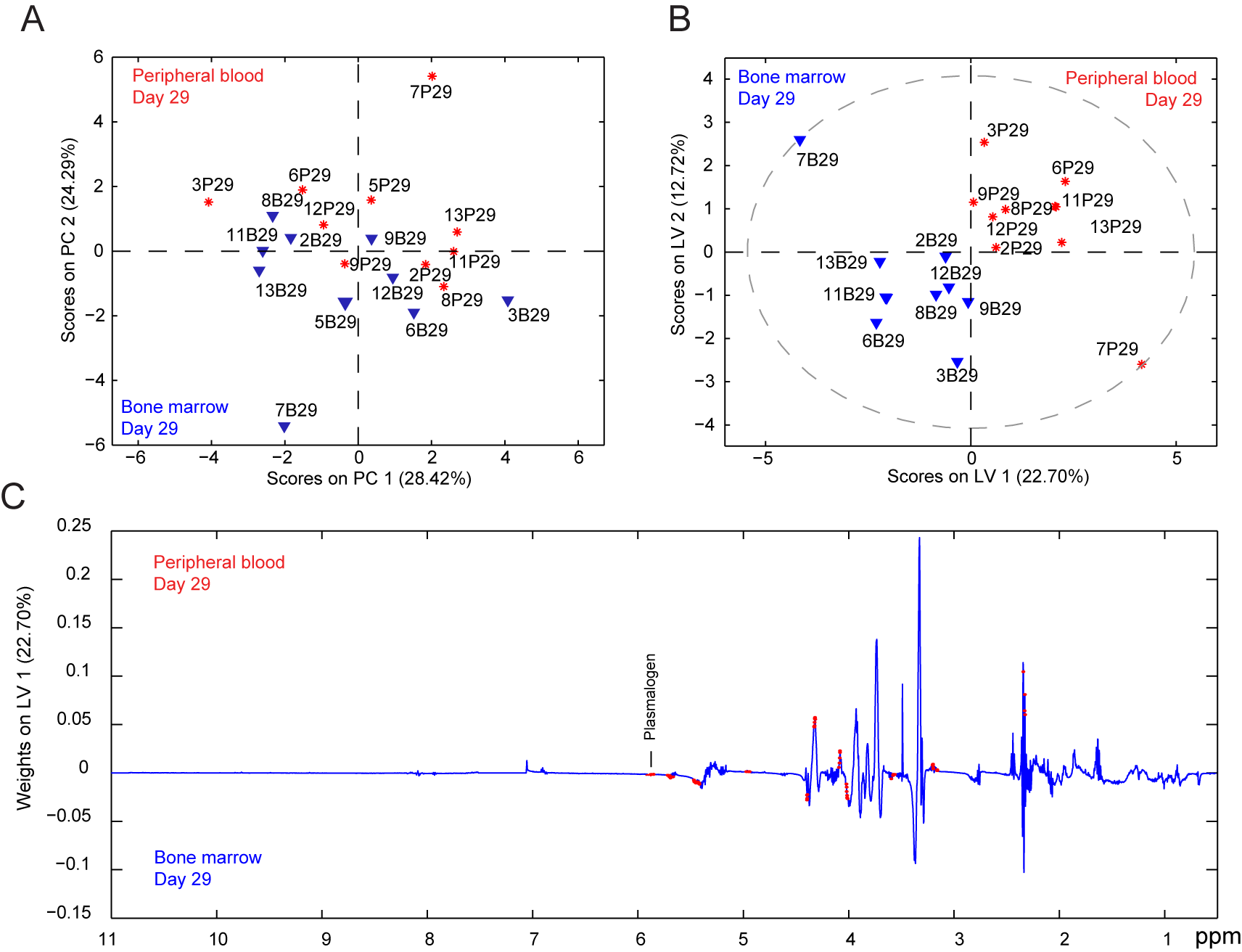


**Figure S5. Multivariate analysis of MRS spectra** **of bone marrow and peripheral blood lipid fractions collected at the end of induction therapy.**

(**A**) mPCA did not show a separation between BM and PB collected on day 29. (**B**) Employing a supervised multivariate analysis, the multilevel partial-least squares discriminant analysis (mPLS-DA) model was built using two classes and two latent variables (LVs). The predictivity of the model was significant (Wilcoxon p ~0.01) and the sensitivity and specificity values were both 100% on cross-validated multivariate analysis DA using ROC curve analysis. (**C**) Corresponding weights plot on LV1 (22.70%) depicts the most relevant discriminatory functional groups for BM (negative weights) and PB (positive weights) samples collected at day 29. Red areas in **C** indicate significantly different regions of the MRS spectra according to a point-by-point nonparametric Wilcoxon Rank Sum Test (WRST; p < 0.05) analysis.


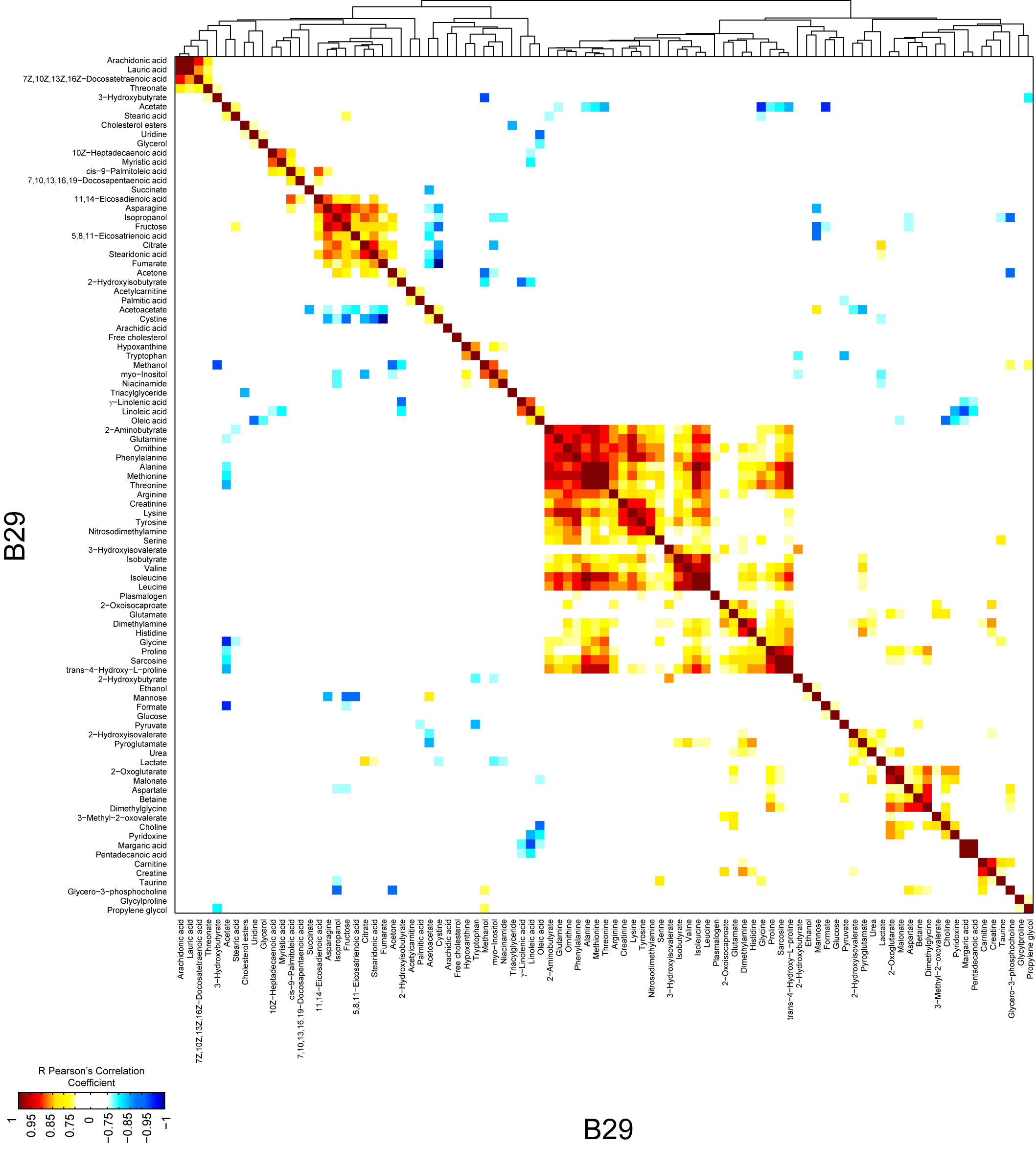


**Figure S6. Heat map of correlations between metabolites in the bone marrow collected at the end of induction therapy.**

Hierarchical clustering analysis of Pearson’s coefficients calculated from the absolute metabolite concentrations (**Table S1**) in BM samples collected on day 29. Only Pearson’s correlation coefficients r > 0.75 or r < ‑0.75 are shown. Pairs of metabolites are hierarchically classified using city-block distance and average linkage clustering methods. Individual correlation coefficients and p-values can be found in **Table S5**.


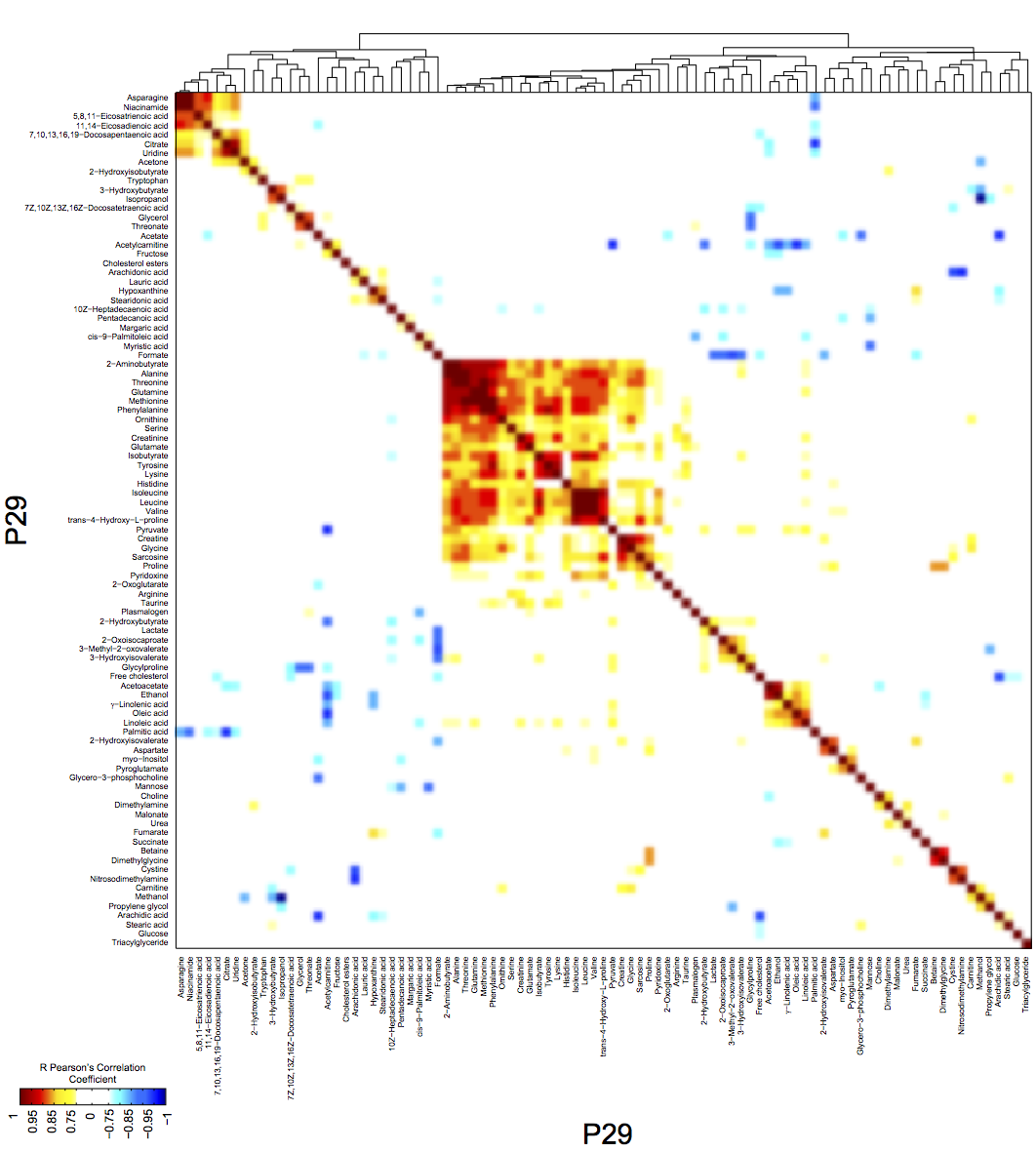


**Figure S7. Heat map of correlations between metabolites in peripheral blood samples collected at the end of induction therapy.**

Hierarchical clustering analysis of Pearson’s coefficients calculated from the absolute metabolite concentrations (**Table S1**) in PB samples collected on day 29. Only Pearson’s correlation coefficients r > 0.75 or r < ‑0.75 are shown. Pairs of metabolites are hierarchically classified using city-block distance and average linkage clustering methods. Individual correlation coefficients and p-values can be found in **Table S6**.


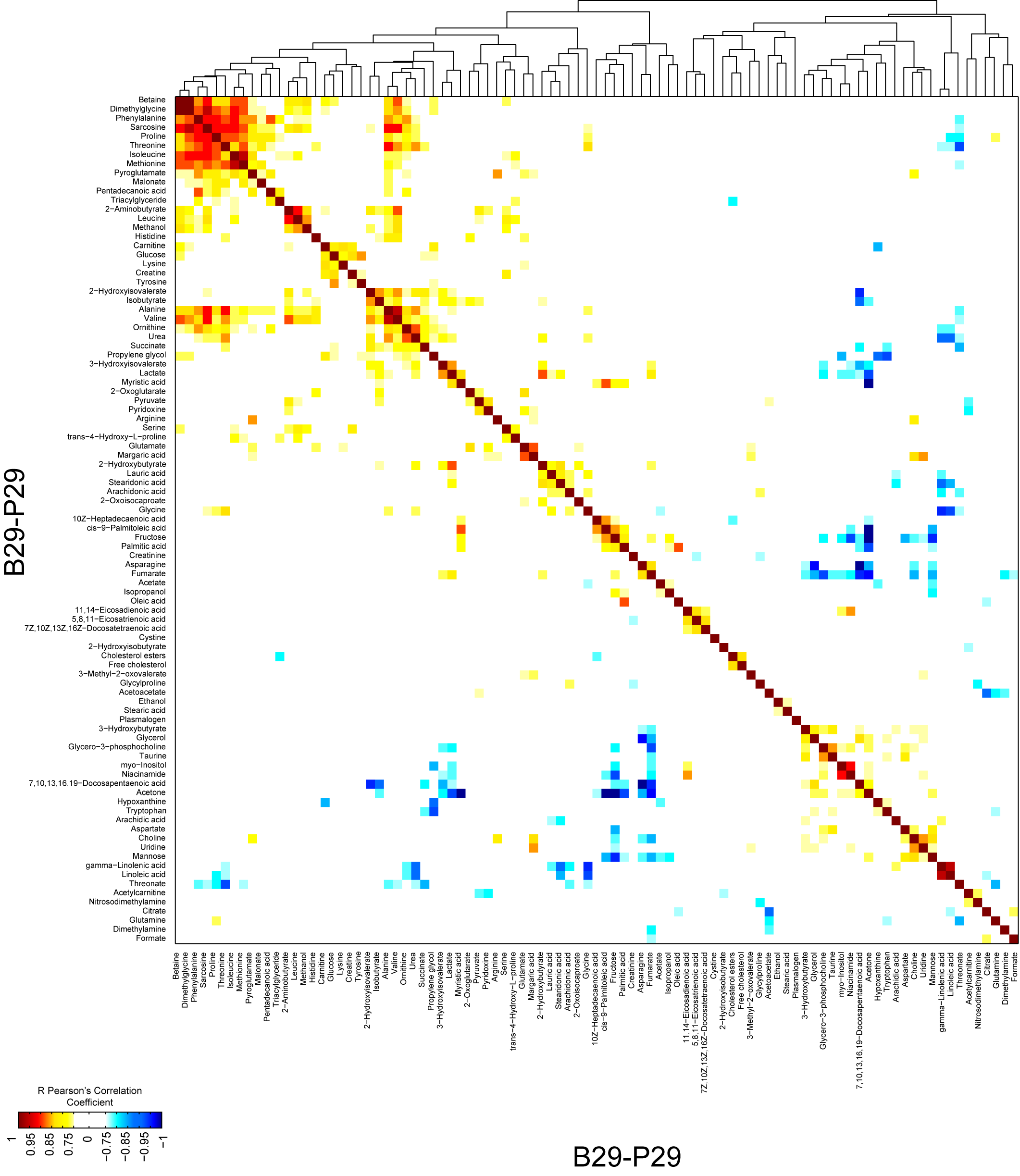


**Figure S8. Heat map of correlations between metabolic differences in bone marrow and peripheral blood samples collected at the end of induction therapy**.

Hierarchical clustering analysis of Pearson’s coefficients calculated from the difference in absolute metabolite concentrations in BM and PB samples (B29-P29) collected on day 29. Only Pearson’s correlation coefficients r > 0.75 or r < -0.75 are shown. Pairs of metabolites are hierarchically classified using city-block distance and average linkage clustering methods. Individual correlation coefficients and p-values can be found in **Table S7**.


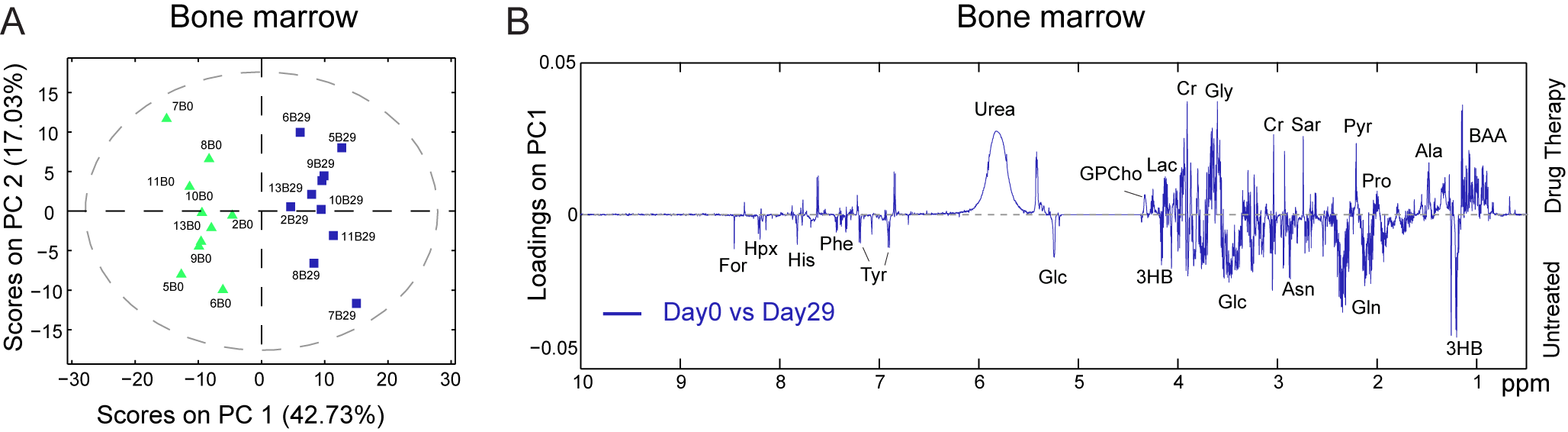


**Figure S9**. **Multivariate analysis of bone marrow polar fractions in response to drug therapy.**

Untargeted mPCA was performed on ^1^H-MRS spectra acquired on BM (**A, B**). (**A**) mPCA scores plot shows a clear separation on PC1 (42.73%) between BM samples collected on day 0 and day 29 (42.73% on PC1). **(B**) Loadings plot for the first principal component highlights the metabolites that discriminate between BM before (negative loadings) and after (positive loadings) therapy. Metabolites are defined in the Abbreviations section.


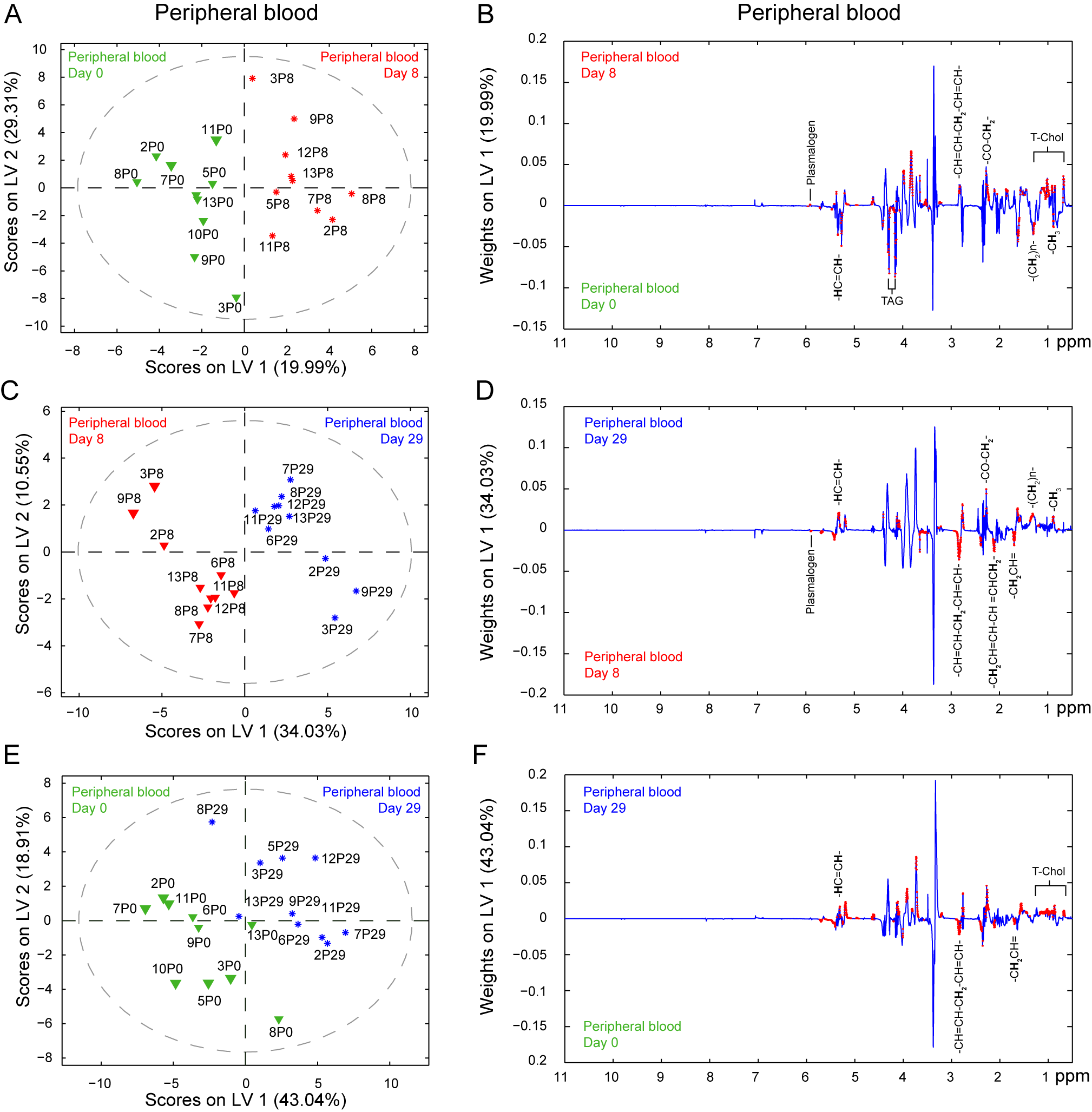


**Figure S10. Multivariate analysis of the peripheral blood lipid fraction in response to drug therapy.**

(**A, C, E)** Score plots from mPLS-DA performed on MRS data acquired on the lipid fraction of PB at diagnosis (day 0), during therapy (day 8), and at the end of therapy (day 29). The model built comparing all three groups was not significant, whereas the models built comparing (**A**-**B)** PB collected on day 0 versus day 8 (19.99% on LV1; 4 LVs, Wilcoxon p ~ 0.003), **(C-D)** PB collected on day 0 versus day 29 (43.04% on LV1; 4 LVs, Wilcoxon p ~ 0.017), and (**E**-**F**) PB collected on day 8 versus day 29 (34.03% on LV1; 2 LVs, Wilcoxon p ~ 0.001) were all significantly validated. Sensitivity and specificity values on cross-validated mPLS-DA models were all >90%. (**B, D, F)** Corresponding weights plots on LV1 depict the most relevant discriminatory functional groups for PB samples in response to drug therapy. Red areas in **B**, **C**, and **F** indicate significantly different regions according to a point-by-point nonparametric WRST (p < 0.05) analysis.


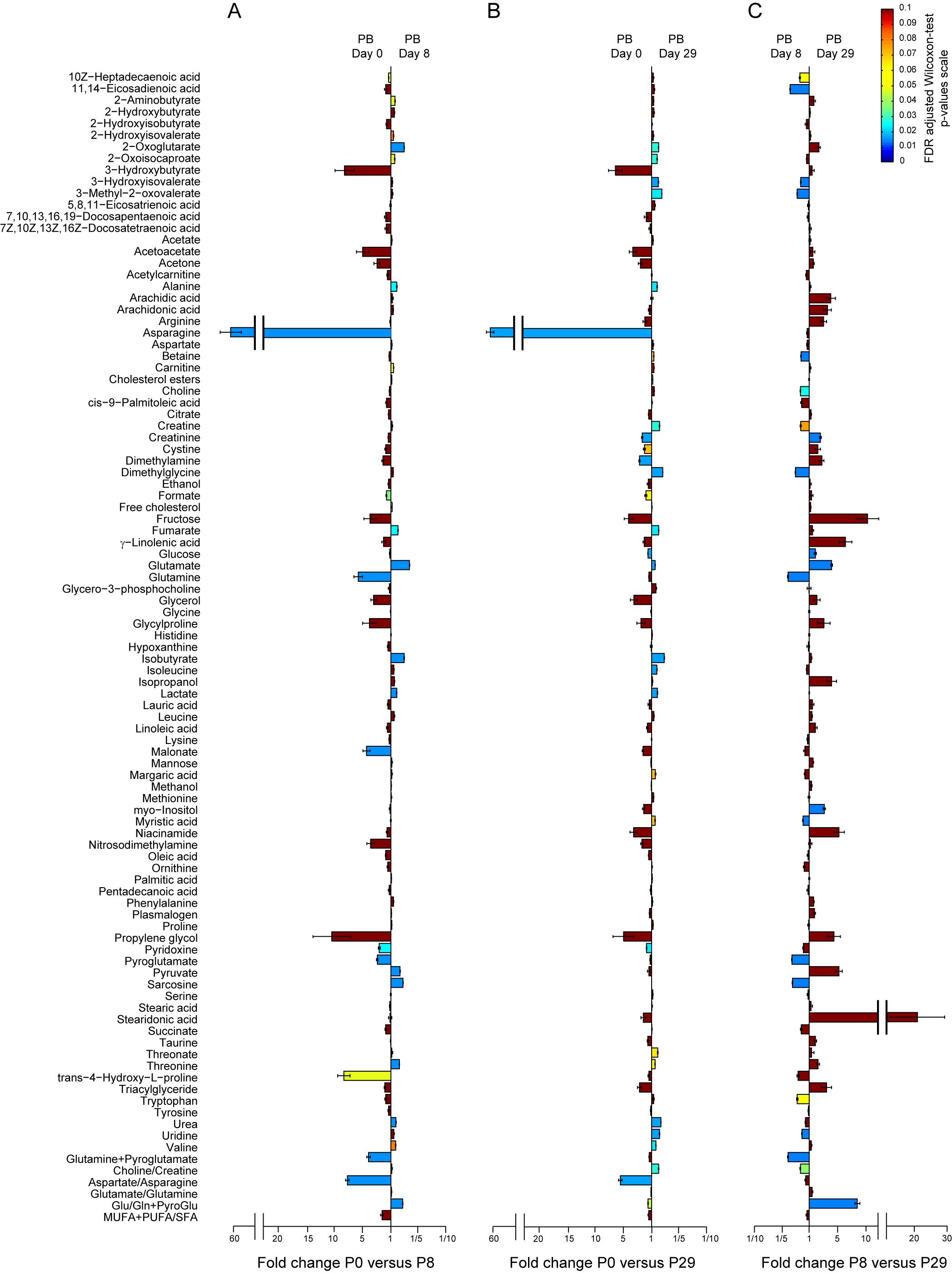


**Figure S11. Targeted** **metabolic analysis of peripheral blood samples at diagnosis and during or after induction therapy.**

Bar graphs show the fold change in absolute metabolite concentrations (mean ± SEM, n = 10 patients) (**SI, Table S2**). (**A**) Change in metabolites between day 0 and day 8. Using WRST, 25 metabolites showed statistically significant differences with pFDR <10%, and 16 with pFDR <5%. **(B)** Change in metabolites between day 0 and day 29. Using WRST, 30 metabolites showed statistically significant differences with pFDR <10%, and 22 with pFDR<5%. **(C)** Change in metabolites between day 8 and day 29. Using WRST, 18 metabolites showed statistically significant differences with pFDR <10%, and 15 with pFDR <5%.


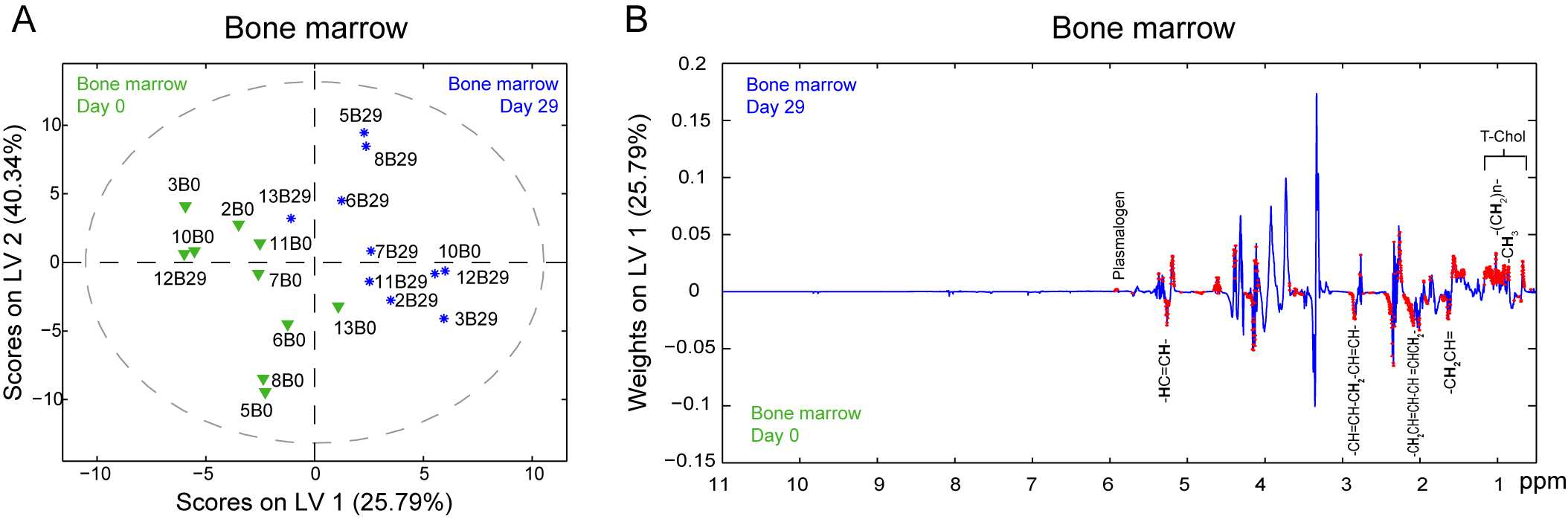


**Figure S12. Untargeted mPLS-DA performed on ^1^H-MRS spectra acquired on the** **whole lipid fraction of bone marrow samples collected at diagnosis.**

Scores plot **(A)** and weights plot on LV1 **(B)** obtained from mPLS-DA performed on full spectra of BM samples collected on day 0 and day 29. The weights plot highlights the most relevant discriminatory functional groups for BM samples before (negative weights) and after (positive weights) treatment. Red areas in **B** indicate significantly different regions according to a point-by-point nonparametric WRST (p < 0.05) analysis.


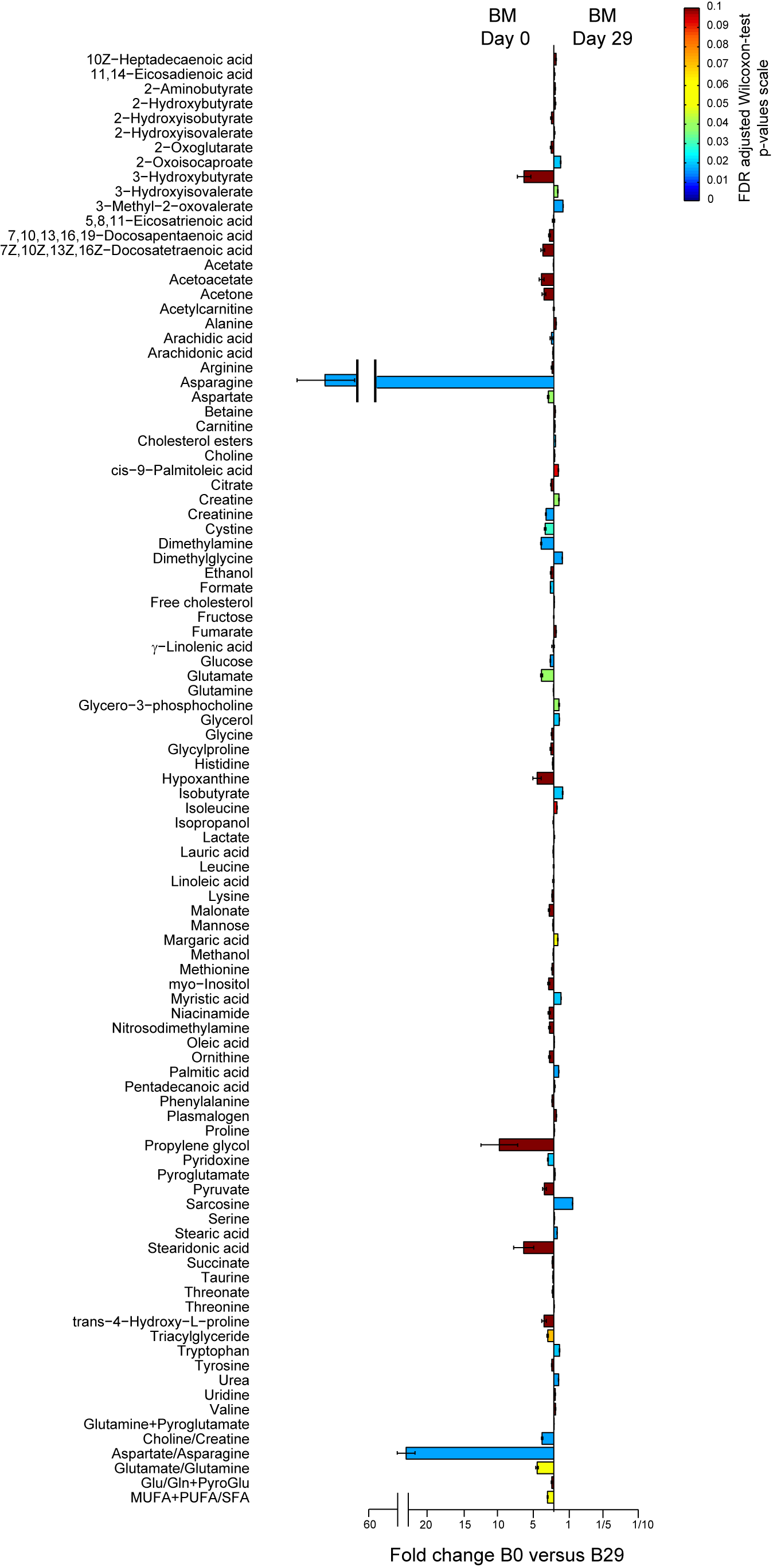


**Figure S13. Targeted** **metabolic analysis of bone marrow samples at diagnosis and after induction therapy.**

The bar graph indicates the fold change in absolute metabolite concentrations (**Table S1**) in BM samples collected on day 0 and day 29 (mean ± SEM, n = 10 patients). Using WRST, 30 metabolites showed statistically significant differences with pFDR <10%, and 26 with pFDR <5%.

**
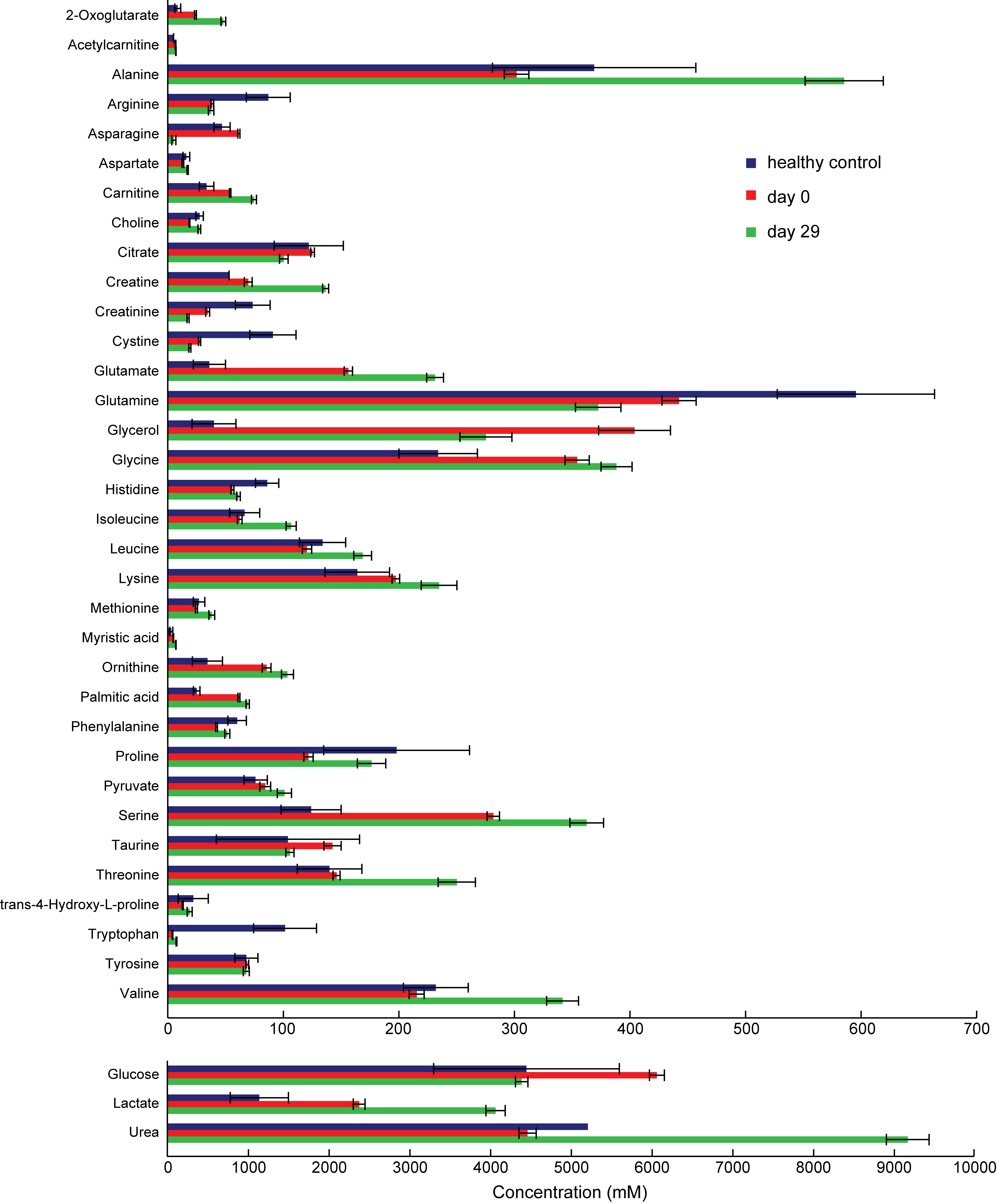
**

**Figure S14. Metabolic concentration from normal pediatric plasma metabolites levels available in the literature.** Data from normal pediatric plasma metabolites levels available in the literature have been compared to the metabolic analysis of peripheral blood samples at diagnosis and during or after induction therapy. Bar graphs show the fold change in absolute metabolite concentrations (mean ± SEM).
